# xDev: a mixed-signal, software-defined neurotechnology interface platform for accelerated system development

**DOI:** 10.1088/1741-2552/adb7bf

**Published:** 2025-03-11

**Authors:** Samuel R Parker, Xavier J Lee, Jonathan S Calvert, David A Borton

**Affiliations:** 1 Center for Neurorestoration and Neurotechnology, Providence VA Medical Center, Providence, RI, United States of America; 2 School of Engineering, Brown University, Providence, RI, United States of America; 3 Carney Institute for Brain Science, Brown University, Providence, RI, United States of America; 4 Department of Neurosurgery, Warren Alpert Medical School of Brown University and Rhode Island Hospital, Providence, RI, United States of America

**Keywords:** neurotechnology, neural interfaces, system development, system integration

## Abstract

*Objective.* Advances in electronics and materials science have led to the development of sophisticated components for clinical and research neurotechnology systems. However, instrumentation to easily evaluate how these components function in a complete system does not yet exist. In this work, we set out to design and validate a software-defined mixed-signal routing fabric, ‘xDev’, that enables neurotechnology system designers to rapidly iterate, evaluate, and deploy advanced multi-component systems. *Approach.* We developed a set of system requirements for xDev, and implemented a design based on a 16 × 16 analog crosspoint multiplexer. We then tested the impedance and switching characteristics of the design, assessed signal gain and crosstalk attenuation across biological and high-speed digital signaling frequencies, and evaluated the ability of xDev to flexibly reroute microvolt-scale amplitude and high-speed signals. Finally, we conducted an intraoperative *in vivo* deployment of xDev to rapidly conduct neuromodulation experiments using diverse neurotechnology submodules. *Main results.* The xDev system impedance matching, crosstalk attenuation, and frequency response characteristics accurately transmitted signals over a broad range of frequencies, encapsulating features typical of biosignals and extending into high-speed digital ranges. Microvolt-scale biosignals and 600 Mbps Ethernet connections were accurately routed through the fabric. These performance characteristics culminated in an *in vivo* demonstration of the flexibility of the system via implanted spinal electrode arrays in an ovine model. *Significance.* xDev represents a first-of-its-kind, low-cost, software-defined neurotechnology development accelerator platform. Through the public, open-source distribution of our designs, we lower the obstacles facing the development of future neurotechnology systems.

## 1. Introduction

Advancements in neurotechnology are shaping the future of medical care for those suffering from neurological dysfunction and injury. However, neurotechnology systems are rarely single component systems. Even simple systems delivering tonic stimulation include a pulse generator, control circuitry, and outputs (e.g. electrodes). The development, evaluation, optimization, and deployment of each subcomponent of a medical device is complex, and combinations of technologies are required to address the complex needs of patients. As materials science has progressed, impressive novel components for neurotechnology systems have been developed (Trevathan *et al* 2019, Steinmetz *et al* 2021, Rachinskiy *et al* 2022, Duraivel *et al* 2023, Parker *et al* 2024). Additionally, improvements in the cost, power, and size of microprocessing technology have enabled closed-loop stimulation control schemes that integrate sensing, signal processing, and neuromodulation into a single system (Morrell 2011, Mekhail *et al* 2020, Lorach *et al* 2023). While these new technologies show promise in isolated laboratory environments, only a select few have successfully completed the regulatory process and achieved sufficient clinical adoption to deliver translational benefit.

There is optimism that bioelectronics are an alternative to drug interventions (Scanlon 2017, Field-Eaton and Pellumbi 2019, Peeples 2019, Khan 2024), however the translation timelines for medical devices mirror the slow and costly development of new pharmaceuticals rather than mirroring the lean, accelerated development of new electronics for the consumer market. The last major neurotechnology translational success was arguably the deep brain stimulator (DBS) developed in the 1980s (delivering electrical neuromodulation to the brain to reduce Essential and Parkinson’s Disease-related tremor) but was not approved by the Food and Drug Administration until 1997 (for Essential tremor) and 2002 (for Parkinson’s Disease) (Gardner 2013). While impressive technologies are on the horizon, overcoming the technical, clinical, and commercial barriers between benchtop successes and bedside therapeutic application requires tremendous investment in time, money and expertise (Schalk *et al* 2024). The inefficiency of bringing new drugs to market is dubbed ‘Eroom’s’ law, given the exponentially increasing cost of drug development in contrast to Moore’s law (Scannell *et al* 2012). The relative success of microelectronic innovation motivates scientists, physicians, and engineers to consider how lessons and strategy from the consumer electronics domain might be applied to confront the complexity of treating neurological disorders, either alone or in combination with pharmacological solutions. From a translational perspective, the efficiency of medical device innovation still has much more in common with pharmacological research and development (R&D) than it does with Moore’s law and consumer electronics.

**Figure 1.**
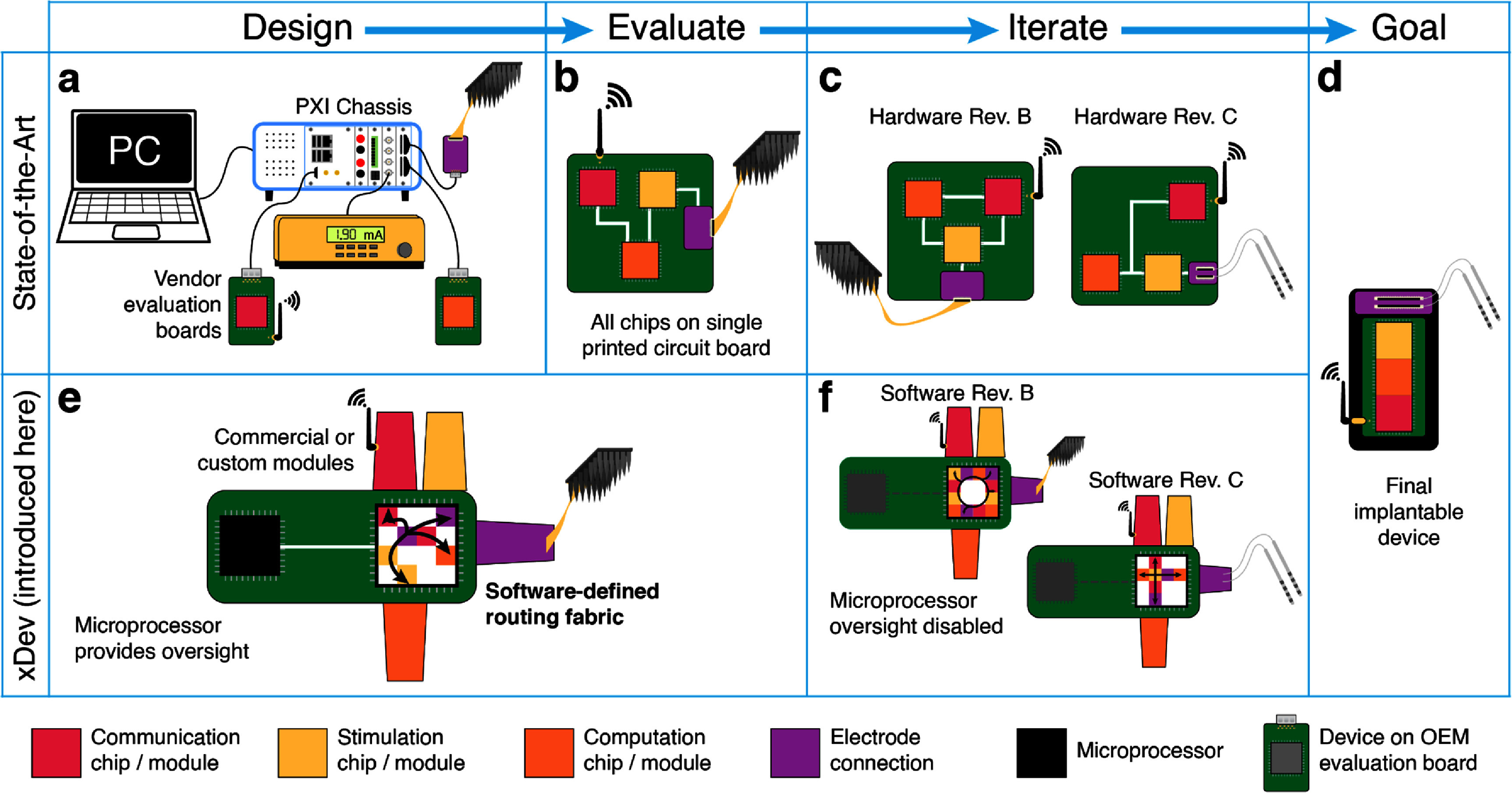
A conceptual overview of the present implanted device design process, and the flexible process using xDev. (a) Vendor-supplied evaluation boards or benchtop devices are subsystems connected using a proprietary backplane, which provides real-time configuration and monitoring to a host computer (PC). (b) Once a system is designed, the component subsystem integrated circuits (ICs) are laid out on a new printed circuit board (PCB) for evaluation. This is a rigid processing step, as changes in the subsystems, layout, or routing are complex. (c) In order to iterate system designs, or correct mistakes, entirely new circuit boards are required, before arriving at the final goal of an implanted device in panel (d). (e) Instead, using xDev, subsystems can either be vendor-supplied evaluation boards, benchtop systems, or custom modules that test a particular layout implementation. The modules are connected using a switch matrix, defined in software. Real-time oversight is provided by a microprocessor. (f) To iterate the system design, modules can be rapidly introduced or removed by updating the software definition for the switch matrix. Here, the microprocessor oversight is disabled, to simulate the modules being on their own PCB.

**Figure 2.**
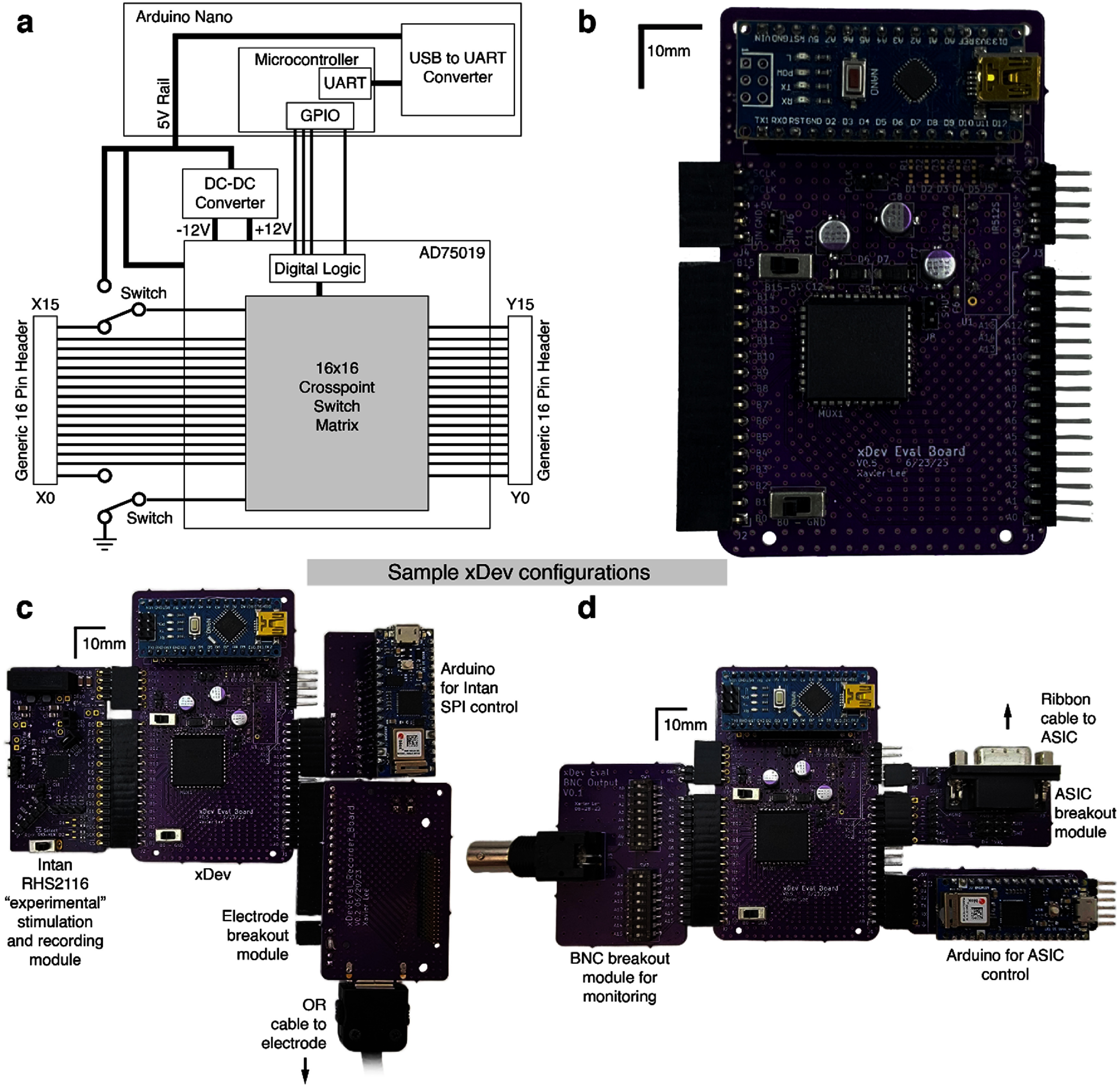
Design and implementation of the initial xDev system. (a) A schematic block diagram of the xDev system. An Arduino Nano facilitates communication to the host computer (PC) over a universal serial bus (USB) interface using a universal asynchronous receiver/transmitter (UART) protocol. Programming of the AD75019 multiplexer is conducted by delivering the desired bitstream over general purpose input output (GPIO) pins, and power is supplied by the Arduino’s 5 V rail. The AD75019 uses digital logic components to configure the 16 × 16 crosspoint switch matrix, which enables arbitrary connection between two banks of 16 pins. (b) A photograph of the initial xDev system as tested here. (c) A photograph of the xDev system in an example deployment to deliver stimulation and record responses using an Arduino Nano 33 IoT and an Intan RHS2116 chip. (d) A photograph of the xDev system in an example deployment to control an application specific integrated circuit (ASIC).

Meanwhile, subcomponents that will comprise next-generation neurotechnology, including amplifiers, stimulation chipsets, wireless telemetry modules, neural signature detection and action algorithms, and neural interfaces themselves are advancing at an exceptional rate (Tseng *et al* 2012, Zhang *et al* 2012, Yakushenko *et al* 2013, Joshi *et al* 2016, Glaser *et al* 2020, Dong *et al* 2023, Lee *et al* 2023, Lee *et al* 2024). Future implantable devices that will provide improved therapy for patients will be systems of multiple interlinked components, requiring fluid communication between subsystems. Today, the development and evaluation of such systems is typically performed using either 1) a highly flexible ecosystem, for example National Instruments data acquisition and control hardware interacting with device components (‘PXI Systems’ 2023) (figure 1(a)), or 2) a purpose-specific ecosystem, for example customized and highly complex circuit boards with predetermined chipsets (figures 1(b) and (c)). Development within a flexible ecosystem necessitates significant redesigns before moving to the final form; development within a highly specialized ecosystem prematurely limits the integration of new technologies over time and adds cost if changes are required. An optimal solution would provide maximal flexibility, simple integration of new components, and require minimal redesign for translation to preclinical and clinical studies.

In this manuscript, we describe our initial demonstration of such a cross-development platform, termed ‘xDev.’ xDev features a software-defined signaling fabric that can be dynamically routed and re-routed to construct novel neurotechnology systems from sub-systems or components (here referred to as ‘modules’) (figure 1(e)). By defining the connections between modules in software, system designers can flexibly swap modules, enabling faster prototyping, iteration, and testing. These modules can exist along a continuum of development: a complete commercial system might control a module containing a single integrated circuit (IC) through xDev. The ability to integrate early-stage, experimental modules with validated commercial systems enables isolated testing of a single device-under-test. Further, engineers can evaluate layouts of custom printed circuit boards (PCBs) as modules, and identify issues with PCB layout decisions (in these modules) early in the design process. More modules can be gradually introduced, and system performance can be assessed at each step (figure 1(f)), as the system moves towards a final implementation (figure 1(d)). This affords system designers the flexibility to rapidly test multiple iterations of their system, including PCB designs and control algorithms.

To this end, xDev is a hardware and software development accelerator platform for rapid prototyping, electrophysiology research, and neurotechnology development. Flexibly integrating sensors, control modules, and end-effector modules on xDev maximizes the ability of neurotechnology device developers to test their tools with versatile interfaces, algorithms, and underlying chipsets, improving compatibility, enhancing cross-functionality, and inspiring new collaborations between technology developers.

## 2. xDev design

When designing xDev, several system requirements were established. These were decomposed into functional requirements, performance requirements, and interface requirements. The following section will discuss each of these design requirements in detail, and the system design steps to meet these requirements.

### 2.1. Functional requirements

The core benefit of xDev is the flexibility to connect assorted modules carrying various signals of interest, ranging from electrophysiology signals (Hz–kHz) (Bronzino 2006) to digital communication (MHz–GHz) (Tarín *et al* 2009, Huang *et al* 2023). Therefore, xDev must faithfully convey a very broad range of frequencies, including high-speed digital signals for control using protocols such as Serial-Peripheral Interface (SPI) or gigabit Ethernet. Since the signals may be differential, routing paths through xDev must present similar impedances to minimize differential effects and impedance mismatches that can introduce common-mode noise. Signals must be routed electronically through a bidirectional switch matrix. Each input and output channel must be capable of being electronically routed to any other set of channels. Electronic routing enables the connections between modules to be defined in software rather than etched into a circuit board, imparting flexibility in development to interchange modules with different pinouts. To define the state of the switch matrix, the xDev system must connect to a host device (e.g. a PC or tablet), and allow users to arbitrarily connect input and output pins. Satisfying these requirements will ensure that the xDev system provides broad utility and benefit to system designers.

### 2.2. Performance requirements

Users of xDev will expect minimal noise and a monotonic frequency response. Noise is an additional, unwanted signal from sources other than the signal of interest. Crosstalk (introduced by electromagnetic coupling of neighboring signals onto a signal of interest), power supply noise, and thermal noise are common examples. Additionally, variations in the amplitude and phase response across frequencies of interest will distort the incoming signal. Therefore, the xDev system must ensure a flat frequency response in the bandwidth of interest (0 Hz–1 MHz). In this bandwidth, xDev must provide sufficient crosstalk attenuation (at least 30 dB) to ensure neighboring signals are not pathologically interfering with each other. Finally, to ensure the conductive surfaces of electrodes connected through xDev do not accumulate dangerous charges, the amount charge of injected when configuring the routing fabric must remain below 30 pCcm^−2^ for common electrode types (e.g. microelectrode arrays, epidural electrodes, and deep brain stimulation electrodes). Meeting these requirements will ensure the xDev system remains electrically transparent, mirroring the performance of an ideal switch.

### 2.3. Interface requirements

The xDev system is designed to enable the connection and control of a diverse range of subsystems, including those with different connectors and pinouts. Therefore, xDev must allow for an extremely broad range of connectors to interface with the routing fabric. Connectors common to clinical applications such as spinal cord stimulation paddles have up to 16 channels (5-6-5, Medtronic, Minneapolis, MN). Therefore, the xDev must have at least 16 bidirectional input and output channels. The signals of interest on these channels may contain both positive and negative potentials, which usually necessitates a split voltage supply. However, for ease of use, the xDev must be powered by a single universal serial bus (USB) connection to the host PC (which provides 5 V power, and a maximum current of 500 mA) and generate the positive and negative voltage rails onboard. Satisfying these requirements will ensure that the xDev system is flexible enough to connect diverse subsystems, while remaining easy to use.

### 2.4. Design solution

To address these requirements, several options were considered. It is possible to construct a matrix of switches using discrete metal–oxide–semiconductor field-effect transistors (MOSFETs). MOSFETs are available to suit low- and high-frequency applications, and conductance through MOSFETs can be electrically controlled using a microcontroller, satisfying the signal range and electronic programming requirements. However, a prohibitively large number of transistors would be required to connect any combination of 16 channels in a discrete implementation, complicating routing and PCB layout. Instead, we used a commercially available analog crosspoint multiplexer IC (AD75019, Analog Devices, Wilmington, MA). The AD75019 provides programmable connectivity between any of its 16 bidirectional input and output channels, respectively (Analog Devices 10/2018), satisfying the number of channels and flexible routing requirements. Exceeding the requirements, more channels can be added by cascading multiple AD75019 chips (programming signals are propagated from one chip to another on buffer overflow, so only a single controller is required, and the minimum configuration time increases linearly). However, further consideration must be given to the signal fidelity requirements.

With respect to signal fidelity, the specified minimum −3 dB rolloff frequency of the AD75019 is 20 MHz, which is sufficient for most biosignal applications (Bronzino 2006), but is likely to limit digital communication performance. It is therefore necessary to verify whether high-speed digital communication is possible through this IC. Additionally, the AD75019 uses complementary metal–oxide–semiconductor (CMOS) switches. Beyond their non-zero impedance, other nonidealities of CMOS switches require consideration in mixed-signal applications. Capacitive coupling arises between signal lines, causing frequency-dependent crosstalk to appear on separate channels and unwanted signal feed-through when switches are turned off. Electrical crosstalk is further increased after the IC is placed on a PCB due to additional parasitic capacitances and inductances. FET switches also possess nonlinear resistance characteristics due to variations in gate–source voltage as the input signal varies, affecting the design considerations of downstream devices. These characteristics will be examined in further detail in xDev Validation.

To control the AD75019 IC, an Arduino Nano (Arduino, Turin, Italy) was included on the board for simplicity of programming and ease of integration into other projects. The Arduino Nano is ubiquitous among hobbyist, research, and industrial applications (D’Ausilio 2012, Sheinin *et al* 2015, Chen and Li 2017, Aguiar *et al* 2020, Taufiq *et al* 2020, Bravo-Martínez *et al* 2023), and can be sourced rapidly and inexpensively. Custom code for the Arduino was written to receive connection instructions from a host PC over a USB link and translate the connections into a bitstream for serial transmission to the AD75019. Once all the bits in the stream are sent, the Arduino toggles the PCLK pin, setting the programmed connections on the multiplexer, satisfying the user programmability requirement. For confirmation, the Arduino reads the received connection information back to the host PC where connection can be verified.

To satisfy the requirements of diverse connectors, a modular approach was taken. To enable connections between xDev and external modules, rows of 16-contact 0.1’ pitch header sockets are included along opposite sides of the main circuit board. These inexpensive and ubiquitous headers are common commodities in electronics prototyping. Then, to facilitate connection between the core and the subsystems, small modules that break out the desired connector can be made with very little engineering effort. Each pin on the header is uniformly spaced, enabling modules to be connected at any point along the span of the header. Multiple modules can share the same header, and can be routed to each other through the multiplexer. The Arduino is socketed to the board using rows of 15-contact headers for easy replacement or upgrade.

The power supply requirement is also handled by the Arduino. The Arduino passes through a 5 V USB supply voltage to power the multiplexer’s digital circuitry and the analog voltage rails via a DC–DC switching converter. Due to the 5 V supply bypassing the Arduino’s onboard voltage regulator, the 5 V rail is directly subject to switching noise from the USB power source. Power supply ripple rejection or coupling is not reported in the AD75019 datasheet (Analog Devices 2018), however including decoupling capacitors close to each power pin of the multiplexer minimizes the effect of power supply ripple. An NMK0512SC DC–DC converter (Murata Power Solutions, Westborough, MA) generates +12 V and −12 V, respectively, to power the positive supply (*V*_DD_) and negative supply (*V*_SS_) pins of the AD75019, setting the specified multiplexer input voltage range to +12.5 V and −12.5 V. No further regulation is provided for these rails, however decoupling capacitors are included to minimize the effect of power supply ripple. Further discussion of the power supply selection can be found in the discussion. The 5 V power supply rail and ground connections are made available to modules through supplementary connections.

A schematic representation of the xDev system is shown in figure 2(a), and a photograph of the xDev board as constructed is shown in figure 2(b). The constructed board is capable of interfacing with diverse subsystems through its general-purpose input/output connectors. Example deployments are depicted in figures 2(c) and (d), which display systems for control of and recording from an Intan analog front-end and an ASIC, respectively. While it can be anticipated from the specifications of the component devices that the xDev system will satisfy the performance requirements, it is important to verify complete system performance to understand the signal quality implications of this system design. We present extensive verification of the xDev system in the following section.

## 3. xDev verification

### 3.1. Differential impedances are well matched over a broad range of frequencies

**Figure 3.**
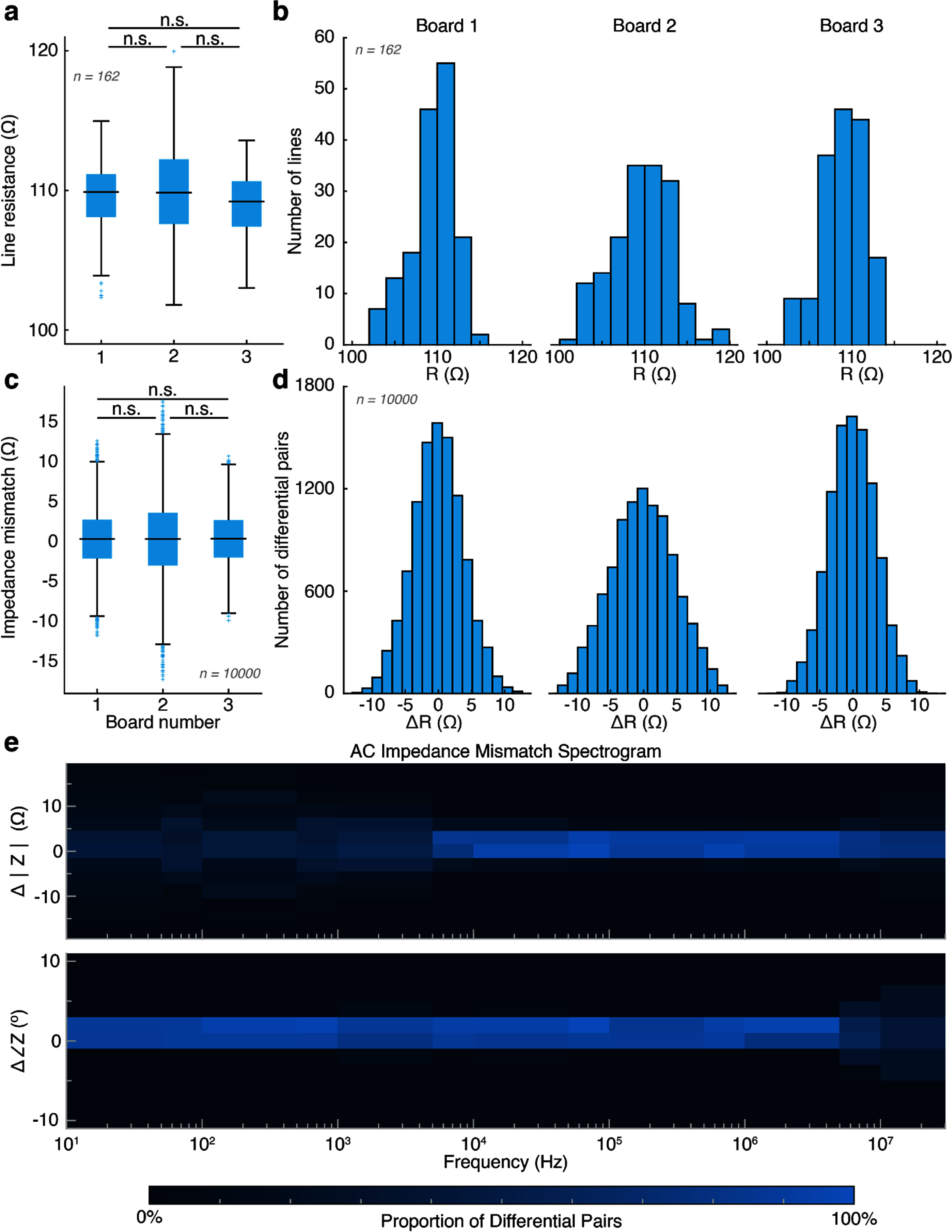
The xDev system demonstrates low direct current (DC) and alternating current (AC) impedance. For (a)–(d): Note, the multimeter uses a 5 V test voltage to determine DC impedance. (a) The distribution of DC line resistances observed for 3 boards. No significant difference in resistance distribution was found between the 3 boards examined (*p* > 0.05, Kruskal–Wallis test, Tukey–Kramer correction). (b) The histograms of DC line resistances observed for 3 boards. (c) The distribution of DC impedances mismatches computed using a bootstrapping method. No significant difference in resistance mismatch distribution was found between the 3 boards examined (*p* > 0.05, Kruskal–Wallis test, Tukey–Kramer correction). (d) The histograms of DC impedance mismatches between randomly selected lines. (e) An impedance spectrogram showing the distribution of impedance magnitude and phase mismatch between randomly selected lines (*n* = 10 000).

We first set out to evaluate the DC resistance of the xDev connections. Switch matrix configuration files were generated randomly using MATLAB (2023b, MathWorks, Natick MA). Connections were added to a file until one of the gates (*X* or *Y*) had already been included in the file, at which point the configuration was complete (ensuring a random number of active connections per configuration). 50 configurations were generated, resulting in 162 total connections (3.24 ± 1.685 connections per configuration). For each connection, the DC resistance was measured using a benchtop multimeter (34401A, Agilent Technologies, Santa Calara, CA) using the Kelvin resistance measurement method. This process was repeated for 3 xDev assemblies. The mean resistances for the assemblies were found to be (109.40 ± 2.62) Ω, (109.62 ± 3.51) Ω, and (108.94 ± 2.42) Ω. No significant differences in the distributions of resistances collected from the boards were observed using a Kruskal–Wallis test (*p* > 0.05), (figure 3(a)). Histograms showing the distribution of resistances are presented in figure 3(b). These values and distributions are within the expected range specified by the AD75019 datasheet.

To assess the resistance matching between multiplexer channels, a bootstrapping technique was used to create 10 000 pairs of connections. The resistance difference between each of the connections was calculated, and the distributions of differences were compared for each board using a Kruskal–Wallis test (*p* > 0.05). No significant differences were found between the resistance mismatches across all boards (figure 3(c)). Histograms of the resistance differences are shown in figure 3(d).

In differential signaling, impedance matching between each differential line is critical to ensure proper performance. To evaluate the impedance matching between xDev channels, a 510 Ω metal-film resistor separated a 2 Vpp sinusoidal voltage source from an xDev channel under test. The impedance of the trace was measured as the input frequency was varied from 10 Hz to 30 MHz in 14 steps (2 steps per decade). The first 16 randomly-generated connections described above were used for this analysis. To simulate differential pairs, 10 000 connection and frequency combinations were generated. The impedance magnitude and phase difference between these differential connections was calculated at the randomly selected frequency. Magnitude and phase spectrograms are shown in figure 3(e). Magnitude and phase differences centered around 0 Ω and 0 degrees, respectively. Differential impedance magnitudes for frequencies below 5 kHz were more varied than for frequencies above 5 kHz, where the differences in impedance magnitude were much smaller. Contrastingly, the difference in phase shift introduced by each line in the differential pairs remained very small from 10 Hz to 5 MHz, then became larger and more diverse at higher frequencies. These properties identify a large range of frequencies (between 5 kHz and 5 MHz) where differential signaling lines will experience minimal differences in impedance magnitude and phase, ensuring each polarity of the differential signal arrives with similar magnitude and timing. While transmission is optimal in this bandwidth, the magnitude of the variations introduced outside of this range are expected to introduce a negligible impact.

### 3.2. CMOS architecture introduces impedance asymmetry: an important consideration in system design

**Figure 4.**
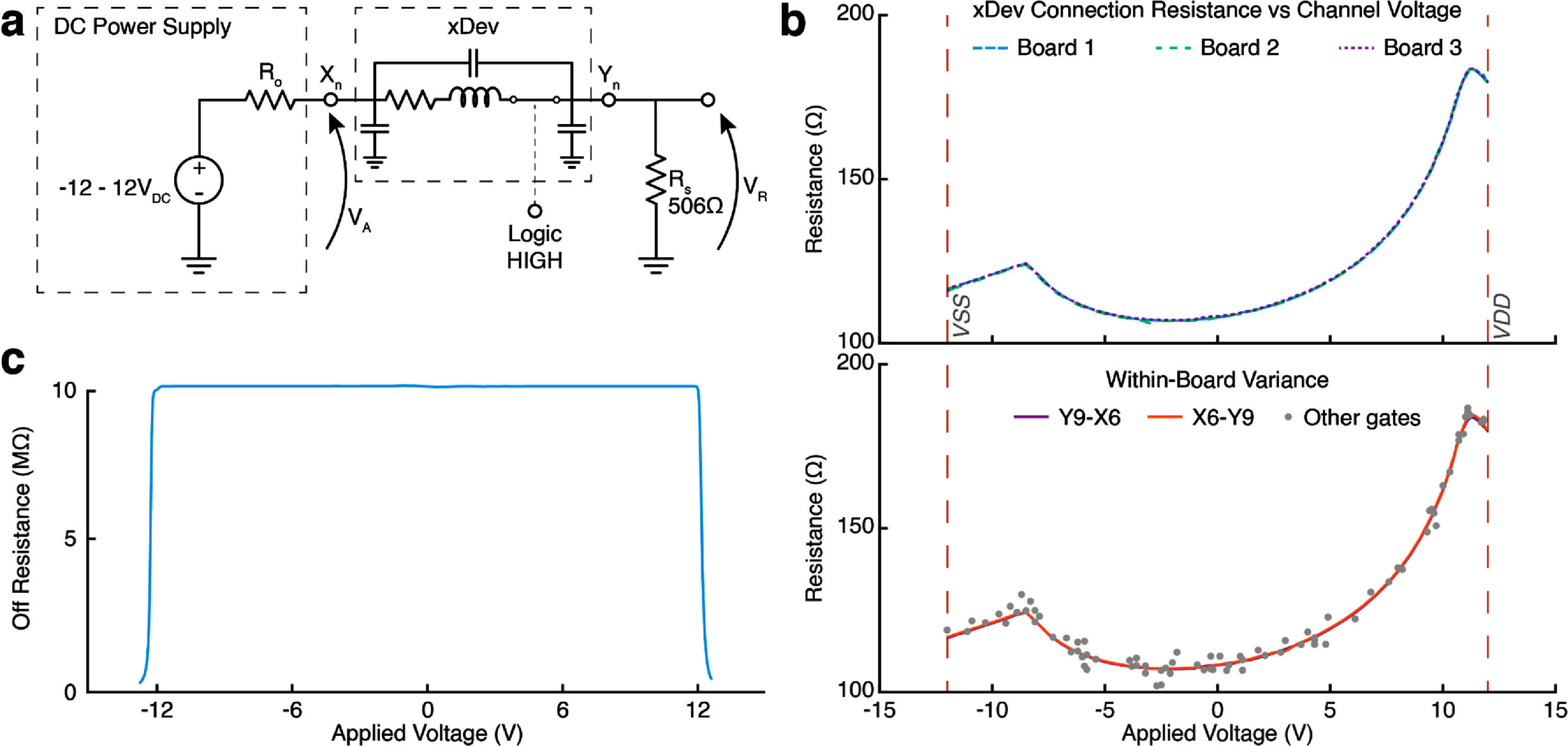
DC impedance flatness performance and off-state resistance. (a) A circuit diagram showing the test circuit used to measure the resistance flatness for an xDev trace. When measuring in the reverse direction, the voltage source was connected to the *Y* gate and the resistor was connected to the *X* gate. (b) (top) Resistance-flatness curves for 3 boards. (bottom) Resistance flatness curves for a single connection on a single board, measured in the forward and reverse settings. Random samples are drawn by randomly establishing single connections on the board. (c) Off-state impedance measurements for an xDev trace.

When CMOS switches are in the on-state, the signal traverses the conducting channel between the drain and source of the transistors comprising the switch. The simplest topology requires an NMOS and PMOS transistor connected in parallel. The inverted biasing voltages applied to the respective gates control the resistance of their respective channels, guaranteeing conduction when both positive and negative inputs are applied. The potential difference between the gate and both the source and the body of the transistors primarily controls the resistance, which varies over the input signal range. Drain-source variations will also affect the channel resistance due to channel length modulation. As both of these effects are non-linear, varying the input signal voltage changes the on-state resistance of the effective channel non-linearly (Ong *et al* 2000, Horowitz and Hill 2015). Two tests were performed to quantify this effect. First, a DC voltage was applied directly to one of the ‘*X*’ inputs/outputs with a current-limiting resistor connecting the ‘*Y*’ input/output to ground. The current through the switch was calculated using the voltage across the current-limiting resistor while the voltage applied to the *X* connection was swept from −12 V to +12 V (figure 4(a)). This allows calculation of the multiplexer connection resistance using Ohm’s Law. In the second set of tests, the test voltage was applied to the *Y* port, and the same resistor connected the *X* port to ground. In both tests, an *X*–*Y* connection was randomly selected to be swept across the entire voltage range, and further randomly selected channels and voltages were tested to examine channel matching. It is important to note that this testing method does not control for channel-length modulation.

Figure 4(b) demonstrates the non-linear relationship between the line impedance and the voltage on the line. The range of impedance values spanned (106.11–184.65) Ω as shown in the top row, and we observed good congruence (sum squared error (SSE) = 37.7 Ω over the full *V*_DD_ to *V*_SS_ range, (0.299 ± 0.2447) Ω mean absolute error ± standard deviation) across the three tested boards. The relationship between applied voltage and line impedance was not significantly different when voltage was applied on the *X* side or the *Y* side (Mann–Whittney *U* test, *p* > 0.05). Any connections made through xDev will contain this nonlinear element in series, requiring that sensitive designs include strategies to mitigate the resulting nonlinear effects. These could include ensuring the range of recorded biosignals remains within a small voltage range (e.g. neural data with a 100 mV range will at worst see a resistance change of 3.07 Ω if DC-coupled, and only 42.2 mΩ if AC-coupled), or selecting an amplifier with a sufficiently large input impedance to minimize the non-linear effects through voltage division while maintaining the desired feedthrough isolation (Horowitz and Hill 2015).

Channel resistance also has consequences for the power delivery capabilities of xDev. If a low-impedance 5 V source tried to power a device through xDev, even an additional resistance of 100 Ω (approximately observed when 5 V is applied to xDev) would cause the available voltage to drop to 4.5 V at 5 mA, per Ohm’s Law. Connecting multiple channels in parallel lowers the effective resistance, but still runs into power dissipation concerns. Analog Devices specifies a power dissipation per switch of 0.722 mW (Analog Devices n.d.). Routing power to modules through the AD75019 is therefore not recommended.

To test off-state resistance, a 10 kΩ 1% metal film resistor was placed between an xDev input/output and a voltage source (E3631A, Agilent Technologies, Santa Clara, CA) with all gates disconnected. The input voltage was then swept between the supply rails while monitoring both the applied voltage and the voltage across (and thus current through) the 10 kΩ resistor. Knowledge of the DC current and applied voltage permits calculation of the off-state impedance. Figure 4(c) shows the off-state impedance characteristic of an xDev trace. Up to the voltage rails, a flat resistance of 10 MΩ was observed. This resistance falls dramatically if the voltage applied exceeds the analog supply rails of the AD75019 multiplexer chip, forward-biasing the input protection diodes. Unlike the on-state resistance, the off-resistance is symmetrical about 0 V applied voltage.

### 3.3. Connections are established in under 100 ns

**Figure 5.**
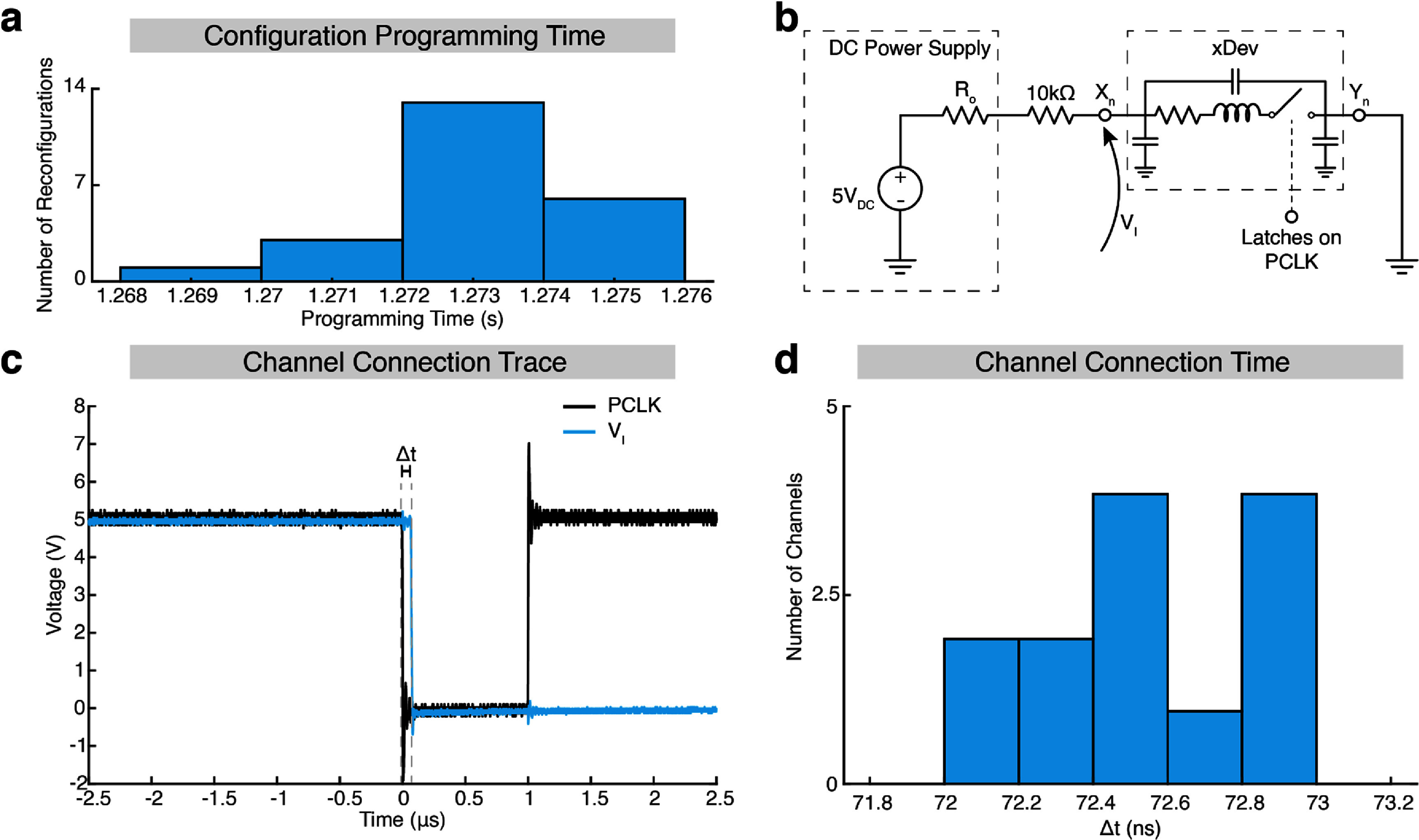
Configuration and switching time were minimized using xDev. (a) A histogram of the time taken to configure the switch matrix using the serial programming interface. (b) A circuit diagram showing the test circuit used to measure the switching time (that is, the time from a configuration being finalized to the connection being made). (c) A time-domain trace showing the configuration being finalized (falling edge of PCLK trace) and the connection being made (falling edge of V_I_ trace). (d) A histogram showing the connection time. The limit of time resolution is 0.2 ns at 5 Gsps.

Using the serial interface to send 256 connections to the multiplexer takes the most time when programming the connections. In the firmware implementation used here, transmitting the data takes between 1.268 and 1.276 s (figure 5(a)). After transmission, the serial protocol requires that the PCLK signal be dropped low, after which the AD75019 updates its internal connections. A delay separates the falling edge of PCLK and this updated connectivity, a quantity crucial to designers for navigating safety, sequencing, and timing of their device.

To measure this delay, an xDev connection was pulled up on one side to 5 V by a 10 kΩ resistor (figure 5(b)). The other end was connected to ground. When the xDev channel connects, the voltage falls from the rail to the divided voltage between xDev’s DC resistance and the pull-up resistor. When disconnected, the voltage floats back up to the rail. Recording the voltage on the xDev channel and the PCLK line on an oscilloscope (MSO-X 3104A, Agilent Technologies, Santa Clara, CA) enabled us to determine the time separation between dropping PCLK and electrical connectivity between the ends of a channel being set (figure 5(c)).

By comparing the times that the signals crossed the 2.5 V threshold, the time taken for the connection settings stored in the digital logic of the AD75019 to become active on the switches could be measured. We measured the connection times for all 16 traces, using the maximum temporal resolution of the oscilloscope (5 Gsps, or 0.2 ns per sample). A histogram of the channel set times is shown in figure 5(d). These times were approximately uniformly distributed between 72 and 73 ns. Examining the time-domain traces enables us to quantify the parasitic impacts of the trace and package capacitance.

Changes in voltage require that parasitic capacitances at the inputs charge or discharge through the resistances apparent to the capacitor. When the connection is made, the apparent resistance includes the xDev channel’s on-resistance, far lower than when the apparent resistance includes the channel’s off-resistance. The discharge curve from the parasitic capacitances follows the known exponential:

\begin{align*}V\left( t \right) = {V_o}{{\text{e}}^{ - t/RC}}\end{align*}

where ${V_o}$ is the initial voltage, $R$ is the trace resistance, and $C$ is the parasitic capacitance. Variations in *R* across the input voltage make it difficult to identify a precise value for the parasitics with this method, however we can make an estimate by fitting an exponential decay model to the data. Using MATLAB, the transition region during which the parasitic capacitance is discharging was isolated, and an exponential model was fit to the data. The time constant *RC* can be identified by algebraic manipulation of the capacitor discharge equation:

\begin{align*}\frac{{V\left( {{t_o}} \right)}}{{V\left( {{t_f}} \right)}} &amp;= {{\text{e}}^{\left( {{t_f} - {t_o}} \right)/RC}} = {{\text{e}}^{\Delta t/RC}}\end{align*}

\begin{align*}RC &amp;= \frac{{t_0} - {t_f}}{ln[V(t_f)/V(t_0)]} .\end{align*}

The apparent resistance when the trace is connected is assumed to be approximately 100 Ω (though as the voltage on the line changes, this value would actually change, as seen in figure 4(b)), allowing us to solve for *C*. Using the 95% confidence intervals of the model parameters to determine the variance, the parasitic capacitance of the traces was estimated to be (37.04 ± 2.85) pF. This value is sufficiently low to present minimal impact on signal fidelity for the signals of interest in this work.

### 3.4. CMOS architecture does not inject unsafe charges onto standard electrodes

The control voltage applied to a transistor gate is capacitively coupled to the conduction channel. When the switch connects or disconnects, the required change in bias voltage necessities charge transfer from each side of the parasitic capacitor. In the simplest case of parallel PMOS and NMOS devices, the inverted control signals required to bias the switch into conduction each inject charge. Because the signals are inverted, the charge transfer occurs in opposing directions. When the parasitic capacitors and control voltages are asymmetric, a net charge is injected into the conduction channel. This charge is drawn through the apparent impedance, and thus, results in a voltage glitch (Horowitz and Hill 2015, 171–180). Additional charge carriers must be drawn into and from the source/drain terminals to form and release the conduction channel, respectively, contributing to the injected charge (Shieh *et al* 1987).

**Figure 6.**
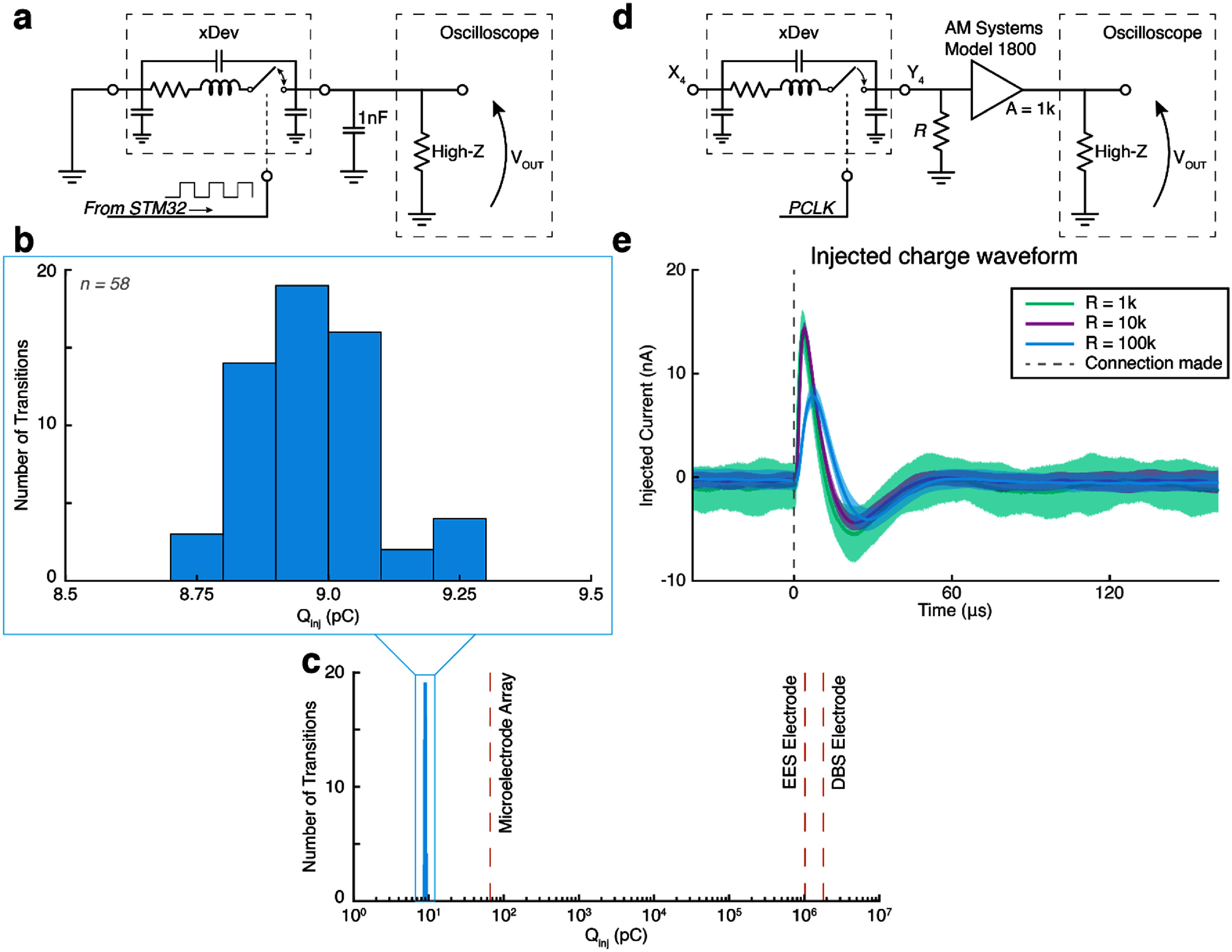
Charge injection performance achieved at safe levels. (a) A circuit diagram showing the test circuit used to assess the amount of charge injected during switching. The gate was opened and closed rapidly, allowing for the charge to accumulate in the 1 nF capacitor, which could be measured as a voltage. (b) The histogram of injected charge during 58 gate cycles. (c) A log-scale showing the measured injected charge, with safe limits for several electrode architectures overlaid in dashed lines (Nordhausen *et al* 1994, Cogan *et al* 2016, De uso n.d.). (d) A circuit diagram showing the test circuit used to measure the charge injection artifact during switching. The gate was closed, and the injected current flowed through the load resistor *R*, representing an electrode impedance. The resultant voltage was amplified, and recorded. (e) The waveforms of the injected currents for each load resistance. Solid lines are the mean, and the shaded region is ±1 standard deviation. The PCLK pulse closed the switch at *t* = 0, indicated by a dashed grey line.

When the channel transitions to the on-state, injected charge can rapidly discharge to ground. When the channel is disconnected, injected charge can only discharge through the very large off-resistance of the switch. By rapidly alternating the connectivity of the switch, the time constant formed by parasitic capacitances and the off-resistance of the switch far exceeds the duration that the switch is kept disconnected. This creates a square wave with amplitude per $\Delta Q\, = \,C\Delta V$, allowing measurement of charge injection when the capacitance is known. To measure charge injection, an STM32 microcontroller was used to rapidly connect and disconnect a single channel faster than achieved using the Arduino controller (figure 6(a)). One side of the channel was shorted to ground, and the other was connected to ground through a 1 nF capacitor, far larger than the parasitic capacitance estimated earlier. Voltage measurements are taken across the capacitor, with an intermediate amplifier to increase signal-to-noise ratio.

The measured voltage waveform across the 1 nF capacitor changed by (9.25 ± 0.12) mV. This gives a charge injection of (8.97 ± 0.12) pC. The distribution of charge injected per switch transition is shown in figure 6(b). The FDA has established a safe limit of charge injected on an electrode as 30 *μ*Ccm^−2^ (Kuncel and Grill 2004, Cogan *et al* 2016). Converting the injected charge values observed here to *μ*Ccm^−2^ is application specific, as it depends on the surface area of the electrode being used. Figure 6(c) instead converts the safe limit of injected charge for three common electrode types into pC for comparison to our distribution. For a microelectrode array of surface area 220 *μ*m^2^ (Nordhausen *et al* 1994), 30 *μ*Ccm^−2^ equates to a charge injection of 66 pC, over 7 times greater than the maximum current injected here. Due to their larger surface area, DBS and epidural electrical stimulation (EES) electrodes can safely handle even more charge. Therefore, we do not anticipate the charge injection induced by mismatches in the FETs of the switch matrix to bring an additional safety concern.

Any charge injected onto electrophysiology channels when switching xDev will present as an artifact voltage. The magnitude of this artifact is dependent on the electrode impedance (by Ohm’s law). We aimed to record the injected current to determine representative artifact magnitudes for electrodes of different impedances. To achieve this, we implemented the test circuit depicted in figure 6(d). When the PCLK signal pulses low, the instrumented channel becomes connected, and the induced voltage across an electrode impedance (from the injected charge) is amplified by an AM Systems Model 1800 amplifier (AM Systems, Carlsborg, WA). After referring the recorded voltage to the input of the amplifier, and dividing by the electrode impedance, the current artifact waveforms displayed in figure 6(e) were observed. These waveforms display a biphasic artifact of approximately 20 nA peak-to-peak. The biphasic nature of the artifact may be due to rise times in the inversion of the control signal required for the PMOS and NMOS devices in the bidirectional analog switch circuitry. A 20 nA artifact induces a voltage ranging from 10 s of *μ*V in low impedance electrodes to almost 1 mV in high impedance electrodes (supplemental figure 1). The charge injection artifact may interfere with recording low-amplitude neural data in the 50 *μ*s following xDev configuration, and limits the maximum speed at which the xDev can be reconfigured during recording. However, in applications where connections are mostly static (at least 50 *μ*s per connection state), or where microvolt-scale signals can be disregarded for 50 *μ*s, these small, transient artifacts may not be detected.

### 3.5. xDev traces show a flat frequency response from 0.1 Hz to 20 MHz

**Figure 7.**
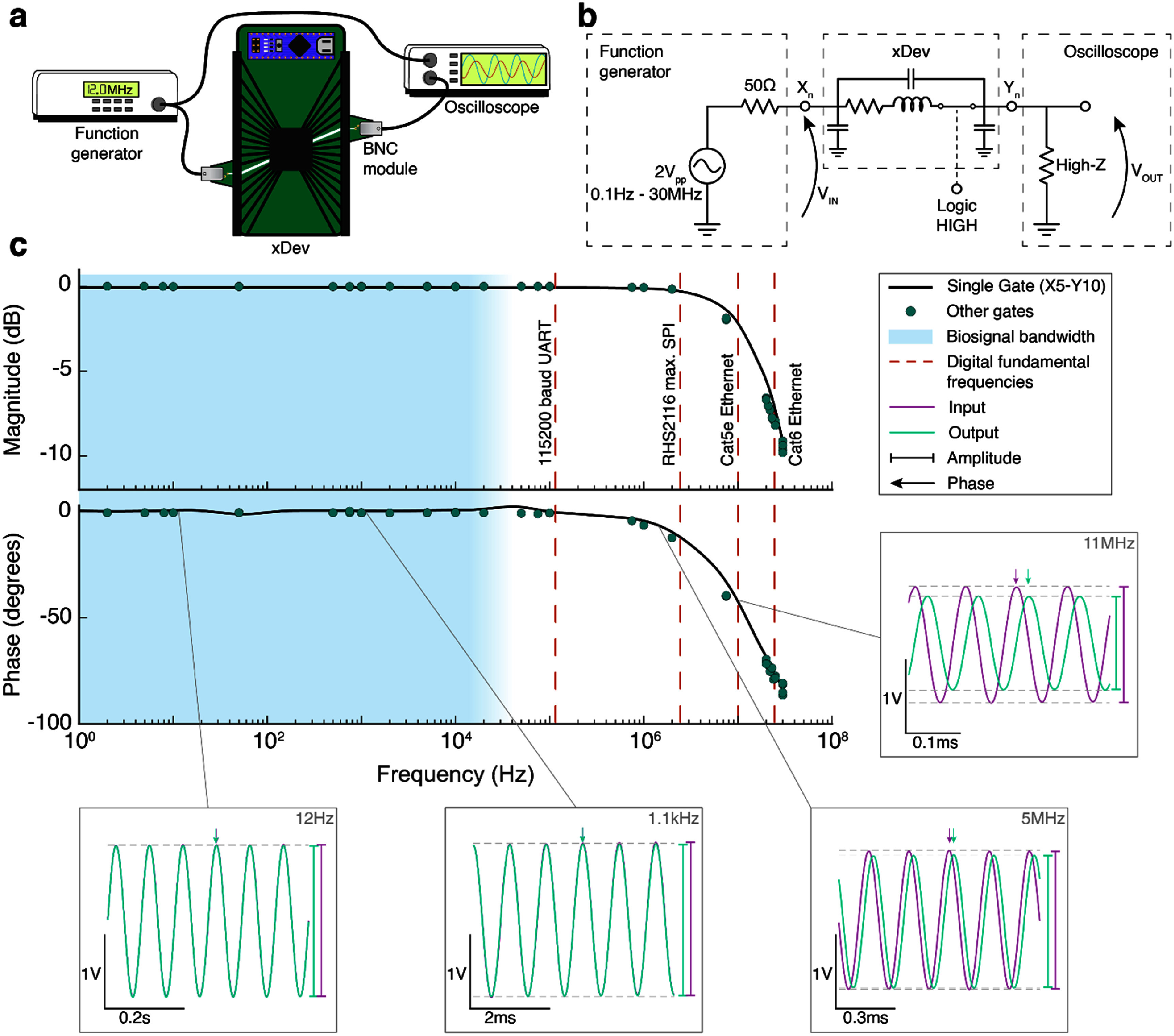
Frequency response of xDev demonstrates minimal attenuation and distortion. (a) A diagram showing the test setup to measure the frequency response. (b) A circuit diagram of the test circuit used to measure the frequency response. (c) A magnitude and phase plot of a single xDev trace, with randomly selected traces overlaid as a scatter plot. The frequency range common for biosignals is shaded, and common digital communication fundamental frequencies are overlaid as dashed lines. Several time-domain examples are inset, to illustrate the change in signal magnitude and phase observed at different frequencies.

It is important to understand the impact that the impedance of xDev traces has on different frequency components of the routed signals. If the impedance of the traces affects certain frequencies differentially, important features of biological signals may be artificially suppressed, enhanced, accelerated, or delayed. To characterize these frequency-specific effects, a function generator was used to deliver 2 V_pp_ sine waves spanning 0.1 Hz to 30 MHz (figure 7(a)). The magnitude and phase of the input and output voltages were measured using an oscilloscope (figure 7(b)), and these values were used to determine the gain expressed by the xDev trace at that frequency using $G = 20{\text{lo}}{{\text{g}}_{10}}\left( {\frac{{{V_{{\text{out}}}}}}{{{V_{{\text{in}}}}}}} \right)$. 4 frequencies per decade were applied to a single xDev connection (X5 to Y10), then random frequencies in the above range were applied to randomly generated connections.

Figure 7(c) presents a Bode plot of the xDev frequency response. The xDev traces present minimal signal attenuation or phase distortion at frequencies below 2 MHz. The two inset time-domain boxes in this range (12 Hz and 1.1 kHz) show the output signal almost-perfectly overlaying the input signal. However, as frequency increases the output signal begins to attenuate, and become delayed compared to the input signal. These effects can be observed in the 5 MHz and 11 MHz inset boxes. This attenuation is the characteristic response of a low-pass filter, such as the one created here by the parasitic capacitance and the resistive elements of the xDev traces. The effect of this response is that high-frequency components of signals may be suppressed. However, the −3 dB corner frequency of the xDev trace is 20 MHz. This is much higher than the typical biosignal frequency range (shaded in blue) (Bronzino 2006). Therefore, since the magnitude and phase of the xDev frequency response is close to 0 dB and 0 degrees in this range, we expect minimal distortion introduced to biosignals. A 20 MHz corner frequency, however, will limit the speed of digital communication, which includes harmonics at odd-multiples of the fundamental frequency. Fundamental frequencies for several common digital communication protocols are overlaid as dashed lines. Lower-speed signaling such as universal asynchronous receiver transmitter (UART) or SPI have fundamental frequencies well within the flat region of the frequency response. However, higher-speed communication such as Ethernet have fundamental frequencies in the rolloff region, and may be affected by the capacitance of the xDev traces. We will now explore the ability of the xDev to route high speed Ethernet signals.

### 3.6. xDev supports high-speed digital signaling

**Figure 8.**
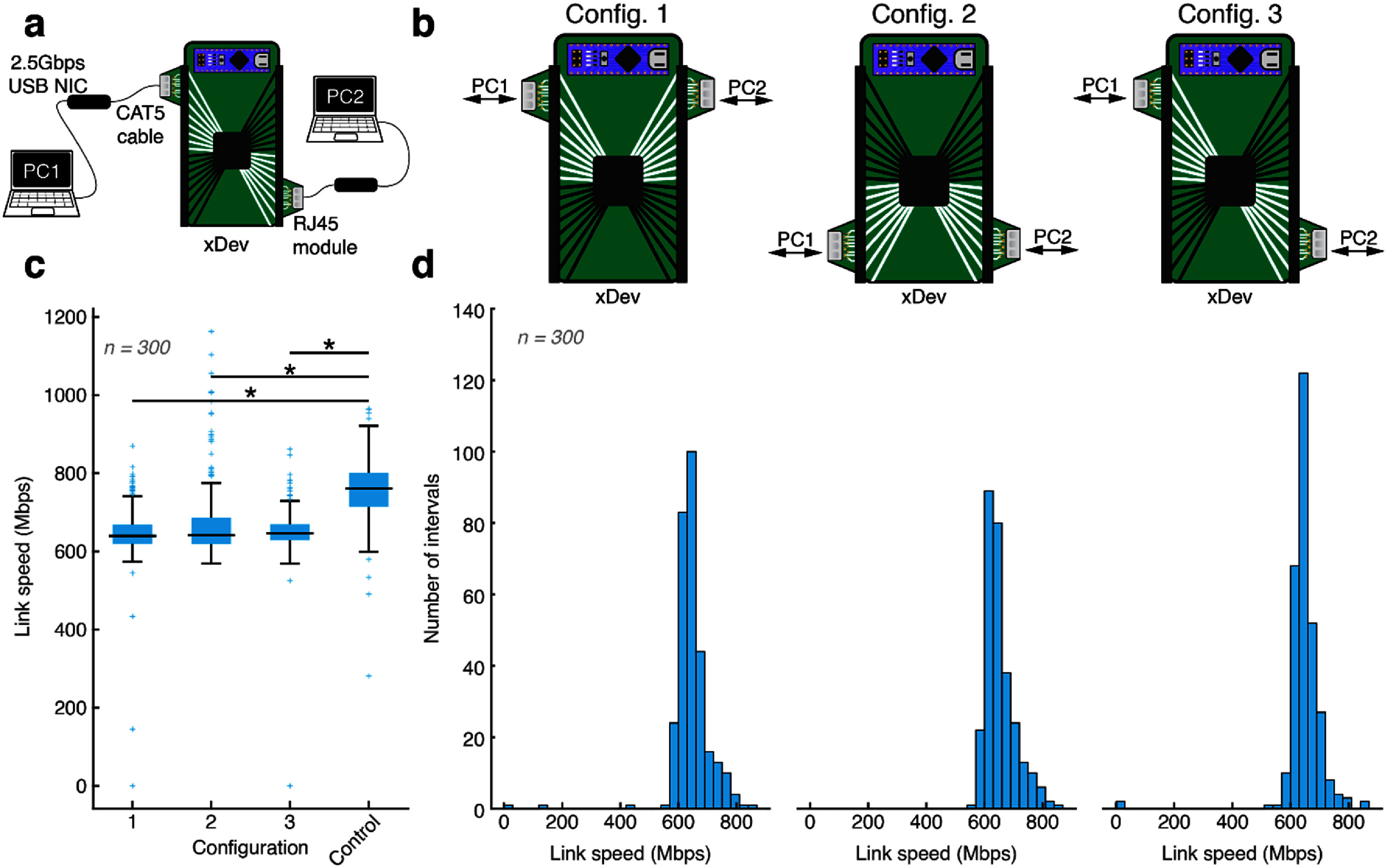
Ethernet network connection delivers high-bandwidth data at sufficient speed. (a) A diagram showing the experimental setup to measure the Ethernet performance of xDev. (b) A diagram showing the different trace configurations tested. (c) A box plot showing the distribution of Ethernet speeds achieved through the three configurations. No significant difference in Ethernet speed was found between the 3 boards examined (*p* > 0.05, Kruskal–Wallis test, Tukey–Kramer correction), however including xDev in the communication path reduced the observed connection speed compared to the ‘no xDev’ control configuration (*p* < 0.0001, Kruskal–Wallis test, Tukey–Kramer correction). (d) Histograms showing the distribution of Ethernet speeds achieved through the three configurations.

Many modern biosignal acquisition and control instruments communicate using an Ethernet interface. Therefore, it is necessary to confirm the ability of xDev to reroute Ethernet signals to a target module while maintaining sufficient link speed. Ethernet breakout modules were used to connect two laptops over a 2.5 Gbps Ethernet network interface through xDev. Each laptop used an external 2.5 Gbps Ethernet network interface card (NT-S25G, Sabrent, Los Angeles, CA) (figure 8(a)). Though the Ethernet interfaces are theoretically capable of 2.5 Gbps communication, the actual transmission rate is negotiated between the network devices based on multiple factors, including the physical layer. One laptop hosted an iperf3 server, while the other connected as a client (Iperf: iperf3: A TCP, UDP, and SCTP Network Bandwidth Measurement Tool n.d.). The xDev was configured to connect the differential pairs straight-through to the other laptop. Three xDev configurations were tested: configurations 1 and 2, where the signals are routed straight through the multiplexer, and Configure 3, where the signals cross over the board, entering on low-numbered *X* inputs and exiting on high-numbered *Y* outputs (figure 8(b)). In all configurations, a connection was established, and 128 kB packets were transmitted for 5 min while data transfer rates were recorded. No significant differences in Ethernet speeds were observed between the three configurations (figure 8(c), Kruskal–Wallis test, *p* > 0.05). The different configurations achieved Ethernet speeds of (645.67 ± 67.64) Mbps, (668.16 ± 85.46) Mbps and (649.52 ± 66.71) Mbps, respectively. Histograms showing the link speed for each packet are presented in figure 8(d). However, when the test was repeated without xDev in the signaling path, higher speeds were achieved (‘Control’, figure 8(c)). This is expected, due to the additional impedance (including the on-resistance and parasitic components) causing insertion loss and reflections. If the interface to xDev was optimized for Ethernet (for example, including passives for transmission line matching), the fidelity of other signals of interest to neural engineers (for example, biosignals) would be impacted. While this difference is statistically significant (Kruskal–Wallis test, *p* < 0.05), the speeds achieved with xDev are sufficiently fast to deliver high-bandwidth data over an Ethernet network.

### 3.7. Crosstalk between traces is substantially attenuated over a broad bandwidth

As signals are routed through the xDev, their close proximity (on the circuit board, and internal to the AD75019) introduces unwanted effects. Through parasitic inductance and capacitance, signals can couple onto neighboring traces, polluting recorded biosignals or digital streams. This is known as ‘crosstalk.’ We set out to examine the extent to which crosstalk affected the traces of xDev.

**Figure 9.**
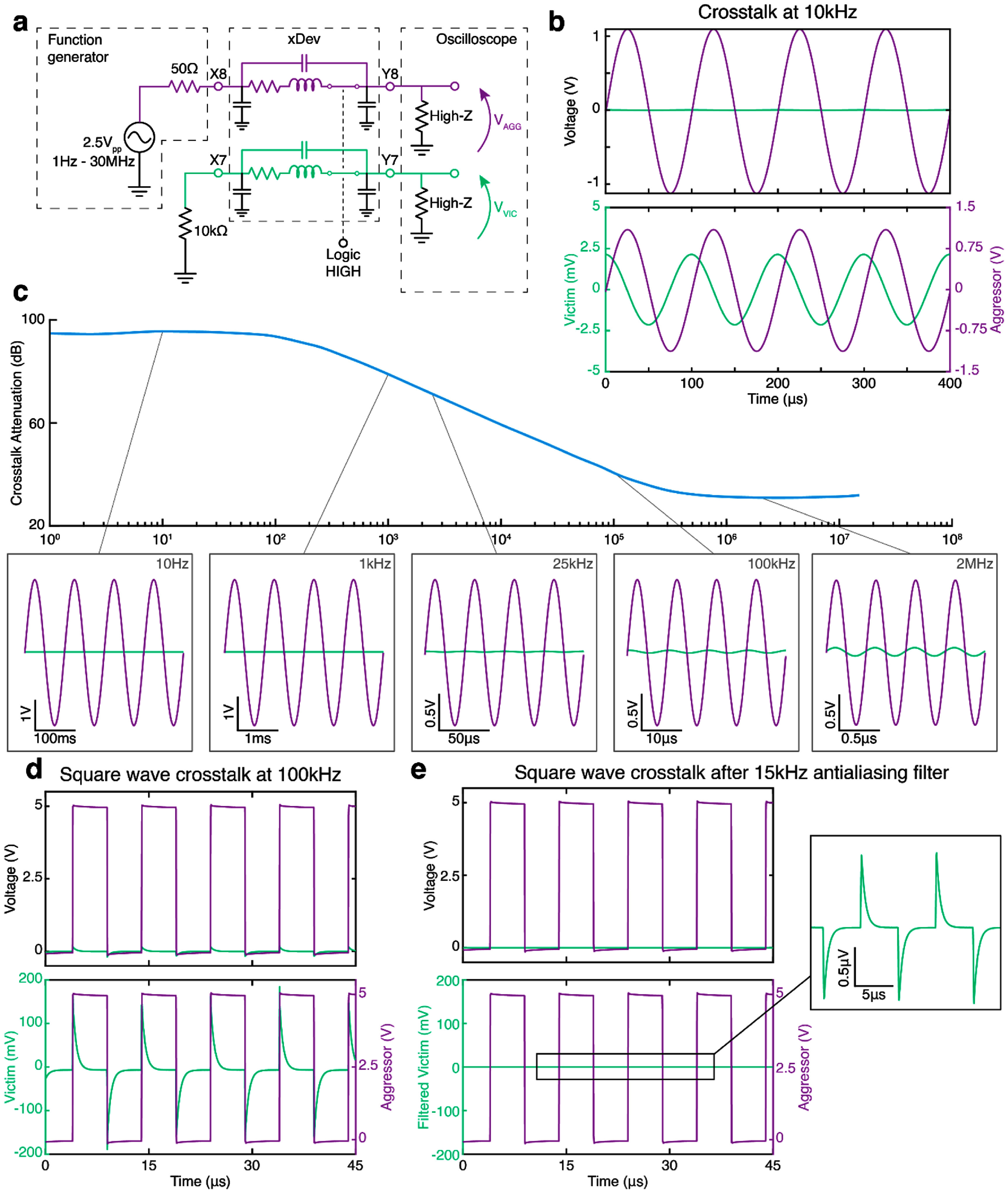
xDev crosstalk was minimal at biologically relevant frequencies. (a) A circuit diagram showing the test circuit used to measure the crosstalk between traces. The aggressor trace is shown in purple, and the victim trace is shown in green. (b) A time-domain trace showing the induced crosstalk at 10 kHz. (c) A magnitude plot showing the crosstalk attenuation between xDev traces vs frequency. Several time-domain examples are inset to demonstrate the changes in magnitude. (d) A time domain trace showing the induced crosstalk from a digital aggressor signal at 100 kHz. (e) A time domain trace showing the effective reduction of crosstalk from a digital aggressor by a 15 kHz antialiasing filter. The crosstalk waveform is reduced to less than 1 *μ*V in amplitude.

We assessed crosstalk using a setup to simulate biological and digital signaling occurring simultaneously through the AD75019. Using the principle of superposition, the biosignal source (representing an electrode, known as the ‘victim’ trace) is replaced with a wire, leaving all impedances in place (figure 9(a)). A 10 kΩ metal-film resistor modeled the electrode impedance of the biosignal source, which was routed through xDev then terminated into a high-impedance, 10× scope probe (300 MHz bandwidth, N2890A, Agilent, Santa Clara, CA) simulating the input impedance of a recording headstage. During crosstalk, the current drawn to and from the parasitic coupling capacitor flows through the parallel combination of these two impedances to ground, developing a crosstalk voltage by Ohm’s Law. Therefore, the induced crosstalk voltage will vary with the impedance of the biosignal source, though only a single impedance value is tested here. A 2 Vpp sine wave acted as an ‘aggressor’ signal, and was routed through xDev on a neighboring trace. The frequency of the aggressor signal was then swept from 1 Hz to 30 MHz (4 steps per decade). The average peak-to-peak voltage on the victim and aggressor lines over 512 aggressor cycles was recorded. At frequencies beneath 25 kHz, an AD524 instrumentation amplifier was used to increase the signal-to-noise ratio of the observed crosstalk. The crosstalk attenuation was calculated by $\alpha = 20{\text{lo}}{{\text{g}}_{10}}\left( {\frac{{{V_{\text{A}}}}}{{{V_{\text{V}}}}}} \right)$, where $\alpha $ is the attenuation in dB, ${V_{\text{A}}}$ is the peak-to-peak aggressor voltage and ${V_V}$is the peak-to-peak victim voltage.

Figure 9(b) shows a time-domain example of the crosstalk observed from the aggressor to the victim when the aggressor signal was provided at 10 kHz. Due to the inductive and capacitive nature of crosstalk, we anticipated that the attenuation would vary with the aggressor frequency. In figure 9(c) we observe this frequency dependence. At low aggressor frequencies, there is very high crosstalk attenuation (mean 94.84 dB for *f* ⩽ 100 Hz), however the attenuation begins to rolloff, reaching 3 dB below its initial value at 250 Hz. The attenuation then decreases exponentially between 250 Hz and 125 kHz before leveling off again (31.83 dB for *f* > 125 kHz). We hypothesize that the flattening of the attenuation at higher frequencies can be attributed to the increasing effect of parasitic inductance reducing that of parasitic capacitance, effectively creating a pole-zero pair and preventing further increase in crosstalk power.

While the low-frequency high-attenuation region has a relatively low bandwidth compared to that of typical biosignals, the crosstalk attenuation performance over the entire frequency range tested will substantially decrease the power of signals on neighboring traces (by over 30 dB). We therefore do not anticipate even high-speed digital signals to interfere with each other, nor simultaneously recorded biosignals. Additionally, a subsequent experiment was performed to assess the impact of aggressor-victim separation on crosstalk attenuation. Channels were randomly selected to act as either the aggressor or the victim, creating separations between 1 and 15 channels. The crosstalk between each pair was measured at 10 Hz, 500 Hz, 7.5 kHz, 25 kHz, 100 kHz, and 2 MHz, using the same method as above. This created a manifold of crosstalk attenuation across an aggressor frequency and channel separation space (supplemental figure 2(a)). Fitting a linear regression model at each frequency identified significant positive correlations between crosstalk attenuation and channel separation at 7.5 kHz, 25 kHz, and 100 kHz (*p* < 0.05, *F*-test, supplemental figure 2(b)). This indicates that the crosstalk effects observed above on neighboring channels can be further minimized through proper channel selection (that is, maintaining maximum separation between high amplitude signals and sensitive low amplitude signals).

Finally, the implications of square aggressor waveforms were examined to model mixed signal routing using xDev. The experimental setup was as described in figure 9(a), except the signal generator produced 50% duty cycle square waves with 0 V offset and frequencies spanning 10 kHz to 5 MHz at 3 steps per decade. An example trace showing the victim and aggressor channels for a 100 kHz aggressor frequency is depicted in figure 9(d). Additional frequencies can be found in supplemental figure 3(a). For all frequencies, a large transient voltage is induced, which rapidly decays after each edge transition. The time constant of the decay was calculated to be 462 ns, and was frequency invariant (supplemental figure 3(b)). While the induced transient is large when considering neural data, it is not large enough to induce a logic level transition for TTL or CMOS digital channels. Therefore, we do not predict crosstalk induced from digital aggressors will produce erroneous data on digital victim channels. Additionally, analog neural data victims are equipped with an antialiasing filter. Typical antialiasing filters present on commercially available neural interface analog front ends specify cutoff frequencies between 2 kHz and 10 kHz (Gunderson 2018, Blackrock Microsystems 2021). To simulate the effects of an analog victim channel, the victim channel was filtered using an extremely lenient 2nd order antialiasing filter with a 15 kHz cutoff. The filtered neural data channel is shown in figure 9(e). The transient voltage spikes have been substantially attenuated, with an amplitude below 1 *μ*V (inset). Therefore, due to the high-frequency nature of the induced transients, we do not anticipate digital crosstalk to be present on neural data lines following antialiasing in typical setups.

### 3.8. Microvolt-scale signals are passed with minimal distortion

**Figure 10.**
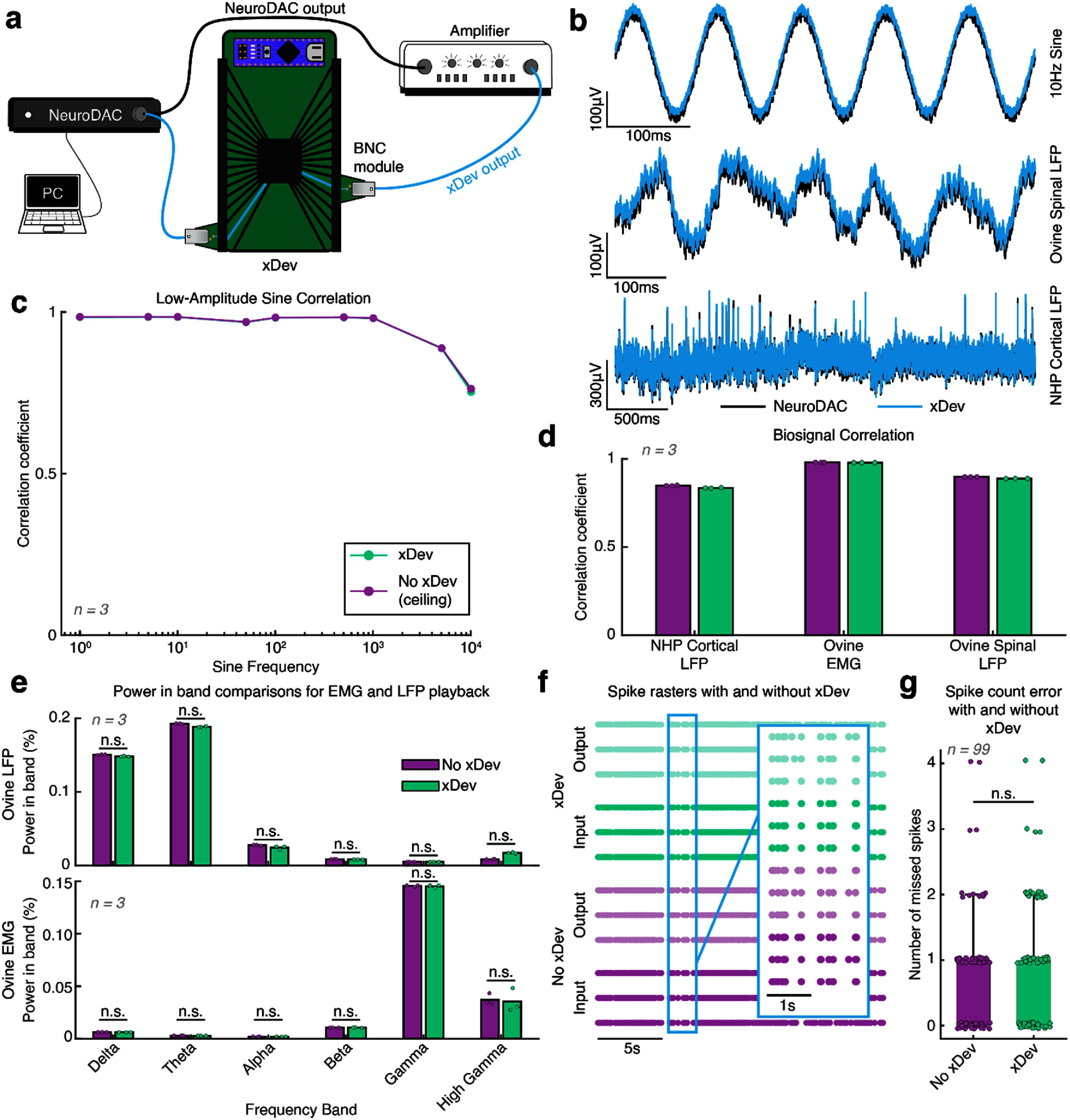
The xDev system faithfully conveys microvolt-scale biosignals. (a) A diagram showing the experimental setup to play back prerecorded biosignals. (b) Example traces for the three biosignals tested. The black trace is measured from the output of the NeuroDAC and the blue trace is measured from the output of xDev. (c) Correlation coefficients between the NeuroDAC and xDev output signals for 300 *μ*V sine waves of various frequencies. (d) Correlation coefficients between the biosignals recorded from the output of NeuroDAC and xDev. (e) Band powers for ovine hindlimb electromyography (EMG) and spinal local field potential (LFP) biosignals with xDev included and excluded from the signal chain. No significant difference in band powers was observed between the ‘xDev’ and ‘no xDev’ conditions for either biosignal (*p* > 0.05, Mann–Whittney *U* test). (f) A raster plot showing the spike times with xDev included and excluded from the signal chain. Each condition was repeated 3 times. (g) A box plot showing the difference in spike counts in 1 s windows with xDev included and excluded from the signal chain. No significant difference in spike count error was found between the ‘xDev’ and ‘No xDev’ conditions (*p* > 0.05, Mann–Whittney *U* test).

To determine the impact of placing xDev in the signal chain, we used an open source biosignal playback device (NeuroDAC, (Powell *et al* 2021)) to play three types of pre-recorded biosignals. The biosignals were extracts of cortical local field potentials (LFPs) from a non-human primate (NHP), or spinal LFPs or intramuscular electromyography (EMG) signals recorded from an ovine model. The signals were played using the MATLAB interface for NeuroDAC. The output of NeuroDAC was bifurcated, with one branch being routed through xDev then recorded following amplification using an AM Systems Model 1800 amplifier (AM Systems, Carlsborg, WA). The second branch was directly amplified then recorded (figure 10(a)). In addition to the pre-recorded biosignals, biosignal-amplitude sine waves were played using NeuroDAC and recorded in the same way.

Example traces from the sine wave and biosignal playback experiments are shown in figure 10(b). Visually, there is a high degree of similarity between the signals recorded with and without xDev in the signaling chain. To quantify the similarity, we computed the correlation between the signals with and without xDev. First, we completely removed xDev from the setup and played the signal directly into both amplifier channels. The correlation between these two signals set the performance ceiling we could expect from xDev (due to non-idealities and imperfect matching between amplifier and recording channels). This is shown in purple in figures 10(c)–(g). Then, we reconnected xDev, repeated the same playback, and computed the correlation between input and output signals. This is shown in green in figures 10(c)–(g). We observed a very small decrease in the correlation coefficient when xDev was included in the signaling chain (−0.0015 ± 0.0032 for sine waves in figure 10(c), −0.0085 ± 0.000 92 for biosignals in figure 10(d)), therefore we expect signals recorded through xDev to accurately represent the original signal.

To compare the functional equivalence of the signals recorded with xDev and the original signals, we extracted common features from these biosignals. For the ovine spinal LFP and EMG, we extracted the power in typical frequency bands using the bandpower function in MATLAB. The frequency bands were defined as reported in the literature (that is, Delta from 0.5 to 4 Hz, Theta from 4 to 10 Hz, Alpha from 8 to 12 Hz, Beta from 15 to 30 Hz, Gamma from 30 to 90 Hz, and High Gamma from 90 to 200 Hz). For each of these bands, we compared the power in the band when xDev was included in the signaling chain and when xDev was not included, as a percentage of the total power of the signal (figure 10(e)). No significant differences were found between the conditions (Mann–Whittney *U* test, *p* > 0.05). For the NHP cortical LFP, we first counted action potential spikes from the input signals using a simple amplitude and latency threshold. Using the same threshold values for all conditions, spikes were then counted in the output signals. The spike times are presented in figure 10(f). The spike error count was calculated by identifying the difference in spikes present in a moving 1 s window (33% overlap, giving 99 windows) of the input and output data, and compared for the xDev and No xDev conditions (figure 10(g)). No significant difference in spike error count distribution was observed between either condition (Mann–Whittney *U* test, *p* > 0.05). These examples support our hypothesis that signals recorded using xDev are functionally equivalent to the original signals.

## 4. Using xDev

We have shown above that xDev meets the requirements defined for the neurotechnology integration platform. We also designed xDev to be easy to use and reconfigure, enabling rapid reconfiguration to integrate diverse neurotechnology systems, and have made the design documents and control code openly available on GitHub (Parker *et al* 2025). This section will outline how users of the xDev system can interact with the system to build their own systems using the xDev platform.

The AD75019 was chosen for this work for its low cost, high channel count, and fast configuration switching. This multiplexer enables arbitrary connections of 16 input and 16 output channels, allowing for flexible integration of arbitrary modules of neurotechnology devices. These external modules are small user-generated PCBs and can serve to connect neurotechnology along a continuum of development, including commercial devices or single chips. For example, to connect an established, complete device to xDev, a simple module that adapts the connector present on the device to a 0.1’ header is required. The pinout of the module is not important, as the signals are all routed through the multiplexer and can emerge in any order. Further, for a single chip, a more complex board that also tests supporting power supplies and passives can be created.

The xDev hardware development platform can be leveraged for a multitude of applications, as the modules are user-defined. For instance, a researcher interested in spinal cord stimulation can develop modules containing a spinal cord stimulation paddle and an implanted pulse generator (IPG) then connect them through xDev. If a different electrode is to be used, the electrode module can be removed, a new module installed, and the routings between the electrode and IPG updated using new firmware. Alternatively, if an IPG designer is interested in comparing performance between an existing commercial IPG and a new stimulation chip, the surrounding system can remain in place, and the stimulation module can be swapped. Any required control signals for the stimulation chip can be added as an additional module, and routed through xDev by specifying the connections in software. Similarly, a system designer selecting between several recording amplifiers can directly compare the performance of each amplifier by keeping the other system components and signals the same, and simply changing the amplifier module they have connected to xDev. Again, if some amplifiers have different pinouts, or require additional control signals, these device-specific requirements can be satisfied by setting the routing in software. This avoids having to design, manufacture, and verify rigid PCBs for each contender device.

When connecting the xDev to the host machine, the Arduino will create a serial COM port for UART communication. Using a custom Python program on the host machine (available at the GitHub link in Data Availability), an xDev connection specification file will be loaded, and sent to the Arduino over the COM port using the pyserial library (‘Pyserial n.d.). This specification file only contains switch combinations to be connected, however the serial protocol used by the multiplexer requires switch status to be sent sequentially for both connected and disconnected switches. To achieve this, the Arduino firmware interprets the connection information and constructs the complete bitstream (for all 256 switch positions). This bitstream is then sent to the multiplexer, and the switch states are finalized by pulsing the PCLK signal. The actions of the Arduino are opaque to the end user.

Alternatively, a custom graphical user interface (GUI) has been developed using the Qt framework to enable visual selection of the connections to be made through xDev (‘Qt n.d.) (supplementary figure 4(a)). The GUI is provided at the GitHub link in Data Availability, and allows users to select the number of multiplexers cascaded together (hence, the bitstream length and layers of available connections) (supplementary figure 4(b)). Users can then click on individual pins on the multiplexers to assign them a net. At least two pins are required to form a net, and the maximum number of pins in a net is limited only by the number of multiplexers in use. The GUI then generates the connection specification, which is sent to the Arduino as described previously. It is also possible to disconnect all signals through a button on the GUI.

The xDev system described in this study allows routing of up to 16 channels, and has shown a data rate of 600 Mbps using an Ethernet connection. However, more complex systems may require additional channels, or a higher data rate. The number of channels available for routing can be increased by deploying more xDev units. Subsequent units can share the same USB link to the host PC by connecting their serial input lines to the serial output lines of upstream devices. This increases the number of available channels, however since each multiplexer is independent, connections that need to cross over to different multiplexers will require manual connection using, for example, jumper wires.

## 5. Case study: using xDev to evaluate the effects of spinal neuromodulation *in vivo*

We chose to deploy xDev in a real-world setting to rapidly conduct two independent *in vivo* experiments: first using a commercial neural interface processor (NIP) (Ripple Neuro, Salt Lake City, UT), then using a common electrophysiology stimulation and recording headstage IC, the RHS2116 (Intan Technologies, Los Angeles, CA).

All surgical and animal handling procedures were completed with approval from the Brown University Institutional Animal Care and Use Committee, in accordance with the National Institutes of Health Guidelines for Animal Research (Guide for the Care and Use of Laboratory Animals). One sheep (female, aged 5 years, weighing 95 kg) was used for this study. The ovine model was chosen for this study as the spine and spinal cord are comparable in size and share many anatomical features with humans, and the use of the ovine model to study the spinal cord has been well established (Marcus *et al* 1997, Parker *et al* 2013, 2018, 2020, Wilson *et al* 2017, Reddy *et al* 2018).

**Figure 11.**
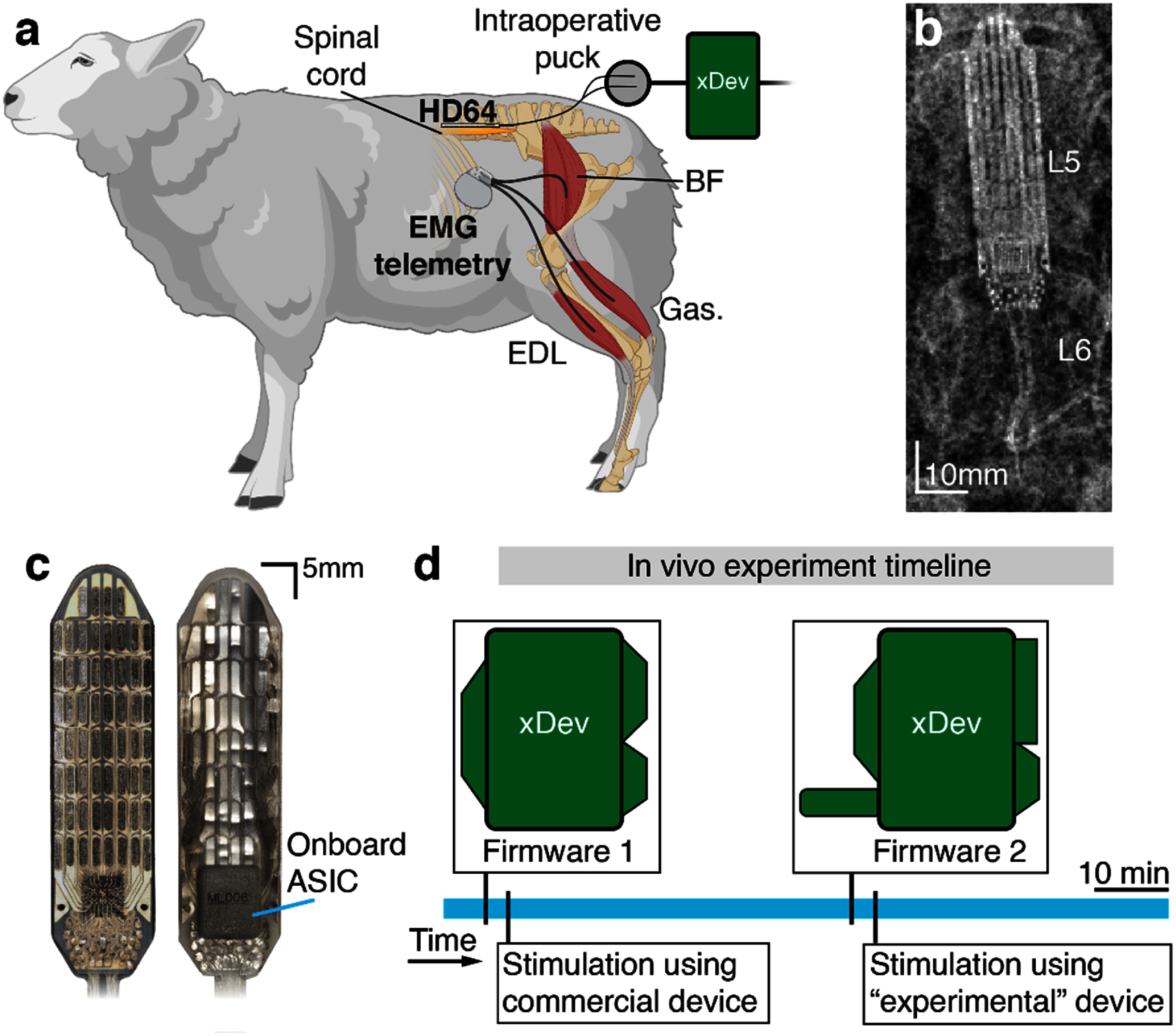
Overview of the *in-vivo* demonstration of system prototyping using xDev. (a) A diagram showing the experimental setup. The epidural paddle array (HD64) was implanted epidurally on the dorsal aspect of the spinal cord, and exposed percutaneously for connection to xDev. The implanted hindlimb electromyography (EMG) telemetry system recorded intramuscular EMG from three lower extremity muscles. The BF is the biceps femoris, the Gas. is the gastrocnemius, and the EDL is the extensor digitorum longus. (b) A radiograph showing the HD64 implanted on the dorsal spinal cord of the sheep at the L5 vertebral level. (c) Photographs of the HD64, showing the 60-contact array (left), and the onboard hermetically sealed multiplexing ASIC (right). (d) A timeline showing the two xDev configurations used in this experiment.

Our sheep EES protocol utilizes an advanced, active, high-density spinal cord stimulation paddle array (HD64, Micro-Leads Medical, Somerville, MA), which requires active power delivery and control signals. Additional details of the HD64, and its implantation protocol, has been described previously (Parker *et al* 2024). Briefly, under propofol-based general total intravenous anesthesia (TIVA), an L4–L6 laminectomy with medial facetectomy was performed. The EES paddle was placed on the dorsal aspect of the spinal cord. Strain relief loops were made, and then the lead-tails were tunneled to the skin, where they were externalized. Reference and ground electrodes (Cooner AS636 wire, Cooner Wire Company, Chatsworth, CA) were secured epidurally and in the paraspinal muscles, respectively. Strain relief loops were made, and the wires were then tunneled and externalized. Intraoperative testing was used to confirm device functionality and anatomical placement. The animals were then recovered from anesthesia and returned to their pens. The anatomy and site of implantation is shown in figures 11(a) and (b). The testing described here occurred during a subsequent anesthetic event, under isoflurane-induced anesthesia.

### 5.1. System descriptions

Two xDev configurations more complex than have been shown thus far were constructed: (1) a baseline configuration, where stimulation and recording was conducted by a commercial NIP (Summit, Ripple Neuro, Salt Lake City, UT), and (2) an experimental configuration, where stimulation was provided by the RHS2116. Both configurations included an advanced, active, high-density spinal cord stimulation paddle array (HD64, Micro-Leads Medical, Somerville, MA) (Parker *et al* 2024) implanted on the dorsal aspect of the spinal cord at the L5 vertebral level (figure 11(b)) and the external HD64 control device. The paddle array control signals were connected to the xDev device through a simple module that converted the native D-Sub 9 connector to 0.1’ header pins, and enabled the HD64 to establish a connection between its input channels and the 60 stimulation contacts (figure 11(c)). Through the software-routing, the signals exited the xDev at the appropriate header pins to be connected to the electrode array through another simple module that adapted the 0.1’ header pins to a Hirose ST60-24P(30) (Hirose Electric Group, Kanagawa, JP), as required by the intraoperative connector block for the HD64. This module combined the HD64 control signals with the electrode signals, but these were routed through xDev to the appropriate target (either the commercial NIP or the RHS2116). To evaluate the ability of xDev to facilitate the rapid changing of neurotechnology devices, we tested these configurations in quick succession (figure 11(d)).

To control the RHS2116, a custom driver was written for an Arduino Nano 33 IoT (Arduino, Boston, MA). The driver allowed stimulation commands to be sent from a host PC to the Arduino over a serial connection, which were then conveyed to the RHS2116 through its SPI. Stimulation trains of varying amplitude, frequency, and electrode could be sequenced by writing experiment design files. These files dictated the stimulation parameters and randomization, and were read by custom Python code on the host PC. The PC-based code handled stimulus duration and inter-train-interval.

### 5.2. Using xDev and a commercial NIP to record spinal electrophysiology

**Figure 12.**
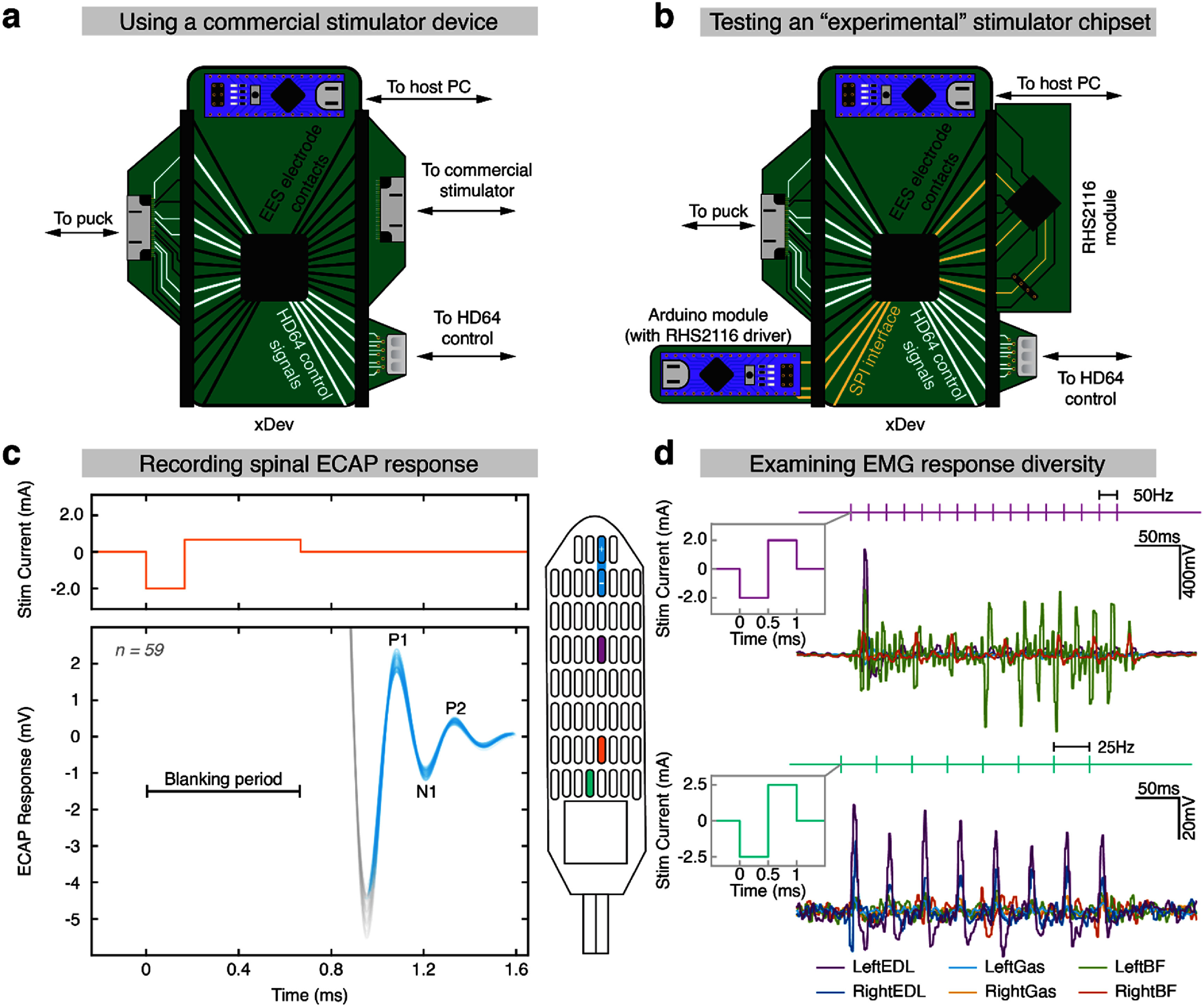
xDev enables rapid system reconfiguration during *in vivo* deployment. (a) The xDev configuration for testing a commercial stimulator device and active epidural electrical stimulation (EES) paddle. Electrophysiology signals and HD64 control signals were routed through the xDev using simple modules. (b) The xDev configuration used to test a custom implementation of the Intan RHS2116 stimulator chip and active EES paddle. A complex module was created for the Intan chip. The chip’s communication signals, electrophysiology signals, and HD64 control signals were all routed through xDev. (c) A fundamental demonstration of recorded stimulation evoked compound action potentials (ECAPs) using xDev. Stimulation was provided using the orange electrode, and the recording was taken from the blue bipolar electrodes. (d) A fundamental demonstration of using xDev to deliver stimulation using a custom stimulator module. In the top panel, stimulation was delivered to the purple electrode at 50 Hz and 2 mA. In the bottom panel, stimulation was delivered to the green electrode at 25 Hz and 2.5 mA.

First, the xDev was configured for the connection to the Ripple NIP. In this configuration, the HD64 control lines and the NIP headstage (Macro + Stim, Ripple Neuro, Salt Lake City, UT) were routed through the xDev to the output module. The output module connected the HD64 to the 0.1’ headers of the xDev (figure 12(a)).

Monopolar, cathode-leading, 3:1 aspect-ratio stimulus pulses with a cathodic phase duration of 167 *μ*s were delivered to a contact on the caudal midline of the spinal cord (orange, figure 12(c)). The current return path was the stainless steel wire implanted in the paraspinal muscles, described previously. Stimulation was delivered at a sufficient inter-pulse interval to avoid depression of the subsequent response (Hofstoetter *et al* 2019). Electrophysiology responses were recorded from two rostral midline contacts, which formed a bipolar recording pair after *post hoc* re-referencing (Parker *et al* 2024). We aimed to recreate the characteristic evoked compound action potential (ECAP) response observed in prior work by our group (Calvert *et al* 2023) and others (Parker *et al* 2013, 2020).

59 stimulation pulses were delivered. The responses recorded through xDev using the bipolar pair were upsampled from 15k samples per second to 1M samples per second using a spline interpolation. The upsampled responses were then aligned such that the first response peak occurred at the mean peak incidence of all responses (figure 12(c)). The responses recorded through xDev matched those presented in prior literature in latency, duration, and amplitude. The characteristic P1 peak (caused by the capacitive current due to initial membrane depolarization) occurs approximately 1.083 ms after the onset of stimulation. The N1 valley (caused by an influx of Na+ ions during the action potentials in the recorded population) is clearly visible, as is the P2 peak (caused by K+ efflux-mediated repolarization of the neurons in the recorded population) (Maruyama *et al* 1982, Struijk 1997, Struijk *et al* 2008, Parker *et al* 2013). This successfully demonstrates the routing of control signals for an active EES array, and spinal electrophysiology stimulation and recording signals through xDev to conduct *in vivo* experiments using commercial neurotechnology components.

### 5.3. Using xDev and an experimental IC

We then sought to configure xDev for the ‘experimental’ IC, the RHS2116. This configuration is the same as above, except the NIP module was replaced with a custom module for the Intan chip, and a module containing the Arduino driving the chip was added. Despite this significant architectural change, using xDev, the routing was updated in seconds by updating the configuration in software.

In this configuration, the HD64 control lines and electrode lines were routed from the output stage through the xDev to the HD64 control device and RHS2116 modules, respectively. The RHS2116 module contained only the IC, a filtered ±7 V power supply (fed from the 5 V USB powering the Arduino), and supporting passives as outlined in the RHS2116 datasheet (Intan Technologies 2021). The module exposed the SPI interface and 12 stimulation channels of the RHS2116. 9 electrode channels was the maximum number of channels possible after 7 of the 16 header pins were consumed by the SPI interface and HD64 control signals. The SPI interface was routed through the xDev to a separate control module containing an Arduino Nano 33 IoT, the device driving the RHS2116.

The goal of this session was to observe the diversity of EMG responses evoked by stimulation controlled by an experimental IC when the stimulation control interface and electrode signals are routed through xDev. To this end, we used our custom driver for the experimental IC to deliver stimulation at various electrode sites, amplitudes, and frequencies. Monopolar, cathode-leading, 1:1 aspect-ratio stimulus pulses were delivered as 300 ms duration pulse trains. Stimulus amplitude was swept from 1500 to 2500 *μ*A and delivered at 25 or 50 Hz. Intramuscular EMG signals were recorded using a wireless telemetry system (L03, Data Sciences International, St. Paul, MN) which had been implanted previously, as described in prior work (Parker *et al* 2024) (figure 12(b)). The inter-train-interval was set to 700 ms. The recorded EMG signals were bandpass filtered between 1 and 2000 Hz.

Example evoked EMG responses are presented in figure 12(d). Stimulation of the purple electrode (more rostral) evoked a strong response in the left biceps femoris muscle (green EMG trace), with some activation of the right biceps femoris (orange EMG trace). The response was phase-locked with the 50 Hz stimulus frequency. When stimulation was applied to the green electrode (more caudal), a weaker, bilateral response was observed in the extensor digitorum longus muscles (purple and dark blue EMG traces). Again, the response was phase-locked with the 25 Hz stimulus frequency. These examples demonstrate the ability to deliver stimuli of known location, amplitude, frequency, pulse width, and duration using an experimental stimulation IC through xDev.

## 6. Discussion

Neurotechnology systems frequently involve the integration of several component subsystems, including sensors, real-time processors, and electrodes. Frequently, these devices are not designed to easily integrate due to several factors, for example, using different connectors or communication protocols. xDev is designed to be a central, reprogrammable signaling fabric through which neurotechnology devices can be interconnected, allowing system designers to utilize a wider range of products to better suit their requirements. In collaboration with benchtop biosignal signal generators, such as the NeuroDAC (Powell *et al* 2021), xDev can enable engineers to assess the performance of an array of candidate devices without the burden of manufacturing, validating, and populating many iterations of PCBs. As demonstrated here, these candidate devices can be commercially available complete systems or single ICs.

Recently, several closed-loop therapeutic applications and research ventures have emerged. Adaptive deep brain stimulation has shown promise as a treatment for epilepsy (Sun *et al* 2008, Morrell 2011, Deeb *et al* 2016), Parkinson’s disease (Little *et al* 2013, Deeb *et al* 2016) and obsessive compulsive disorder (Provenza *et al* 2021). Motor intent has been decoded from M1 population activity recoded using microelectrode arrays in nonhuman primates (Capogrosso *et al* 2016), or electrocorticography grids in human participants (Lorach *et al* 2023) and used to control EES of the spinal cord to restore motor function in nona human participant with incomplete spinal cord injury. Finally, several studies have demonstrated the utility of closed-loop sensory feedback in improving dexterity and balance in upper-limb (Micera *et al* 2009, Zhang *et al* 2015, Schiefer *et al* 2016, Schiefer *et al* 2018, Chai *et al* 2022, Nanivadekar *et al* 2022, Zhang *et al* 2022, Zhang *et al* 2023) and lower-limb (Charkhkar *et al* 2020, Nanivadekar *et al* 2023) prosthetic users, respectively. In all of these applications, a control signal is measured, a real-time controller is applied to titrate appropriate stimulation for the current conditions, and the stimulus is subsequently applied. System designers may evaluate several different options for each of these subsystems as part of the design process. A hardware development platform such as xDev can ease the development process in three major ways: (1) vendor-provided evaluation platforms can be connected together and rapidly replaced with alternative options for initial system development, (2) user-designed PCBs can be tested alongside vendor-provided evaluation platforms to assess the quality of PCB designs, and (3) multiple user-designed PCBs can be connected together to assess full-system performance without freezing the design and etching a single PCB for all functions. This flexibility will likely reduce the time taken between design iterations and the overall cost of future neurotechnology devices.

A contemporary review of the translation of neurotechnologies hypothesized mitigations to translational risk (Schalk *et al* 2024). The authors summarized translational lessons learned from several neurotechnology commercialization thrusts. Their recommendations to optimize development included describing a hypothetical technology platform to improve the practicality and cost of technology and scientific development. They described a general-purpose and modular hardware and software platform to support different types of sensors and stimulators, with sufficient computational capabilities and connectivity to external monitoring and computational resources. Such a platform was hypothesized to be particularly useful during early testing to test multiple approaches and rapidly de-risk a new therapy or technology. Once the proof-of-concept is demonstrated, specialized solutions for the final user can be developed. Following the validation performed here, we believe that the xDev platform as described is an initial offering to fill the gap identified by the authors and the field.

These benefits would not have been directly accessible if xDev had been designed around a field programmable gate array (FPGA). xDev enables dynamic routing of both analog and digital signals, giving system designers the flexibility to work with device pinouts that commingle these domains. While a FPGA exhibits a large number of digital input/output (I/O) pins, analog signals could not be routed through these devices. The effect of this would mean that xDev would have to rigidly demarcate analog pins from digital pins, locking their number and position. It would then be the responsibility of the module designer to ensure that their modules obey these limits. If an application did not require the designated digital pins, but required more analog pins, the digital pins could not be repurposed. To this end, the analog crosspoint multiplexer utilized here demonstrated acceptable signal quality performance for both analog and digital signals, enabling complete routing flexibility.

### 6.1. Study limitations and implications for future research

Although this version of xDev possesses 16 channels, some applications may require more (for example, the HD64 used in the *in vivo* ovine experiments exposes 19 electrode channels and 5 control lines, requiring a minimum of 24 channels to connect to all available electrodes). It is possible to link multiple xDev boards together in parallel, allowing for more input signals to be connected, however this must be done strategically for each application to ensure a routing path exists between xDev boards. Additionally, the power dissipation limitations of the AD75019 chip limits the xDev traces to powering only the lowest-power devices. As such, in this work, 5 V power and ground were shared from the main board to the modules using dedicated connections and jumper wires. A more sophisticated approach is to include additional ground and power connections on the 0.1’ header (either inline or as an additional row), allowing the modules to consume up to 4.7 A (the current limit of 0.1’ headers (Samtec 2023)). This approach will be integrated into future designs. Finally, the use of a two-layer PCB leads to increased trace inductance due to the relatively large separation between the copper trace and the ground plane underneath compared to a four-layer implementation, increasing AC impedance in all cases and inductive coupling when current is carried on a trace. Interruptions in the ground plane also lead to increased AC impedance, and our PCB routing includes many ground plane breaks where ‘*X*’ and ‘*Y*’ traces intersect. These intersections also increase capacitive coupling between channel lines. The use of a four-layer PCB with minimum ground plane breaks and trace intersections is likely to improve signal fidelity at high frequencies.

## 7. Conclusion

In summary, we present xDev, an open-source, low-cost, software-defined development platform for neurotechnology systems. The flexible routing fabric enables experimenters and system designers to rapidly construct, evaluate, and iterate their setup. Following system design and custom development, we conducted extensive benchtop characterization of xDev to examine its performance when carrying signals of interest to neural engineers. Using an open-source biosignal playback device, we identified favorable performance across diverse biosignals, from LFPs to cortical spiking. Finally, an acute *in vivo* deployment demonstrated the utility of xDev by rapidly deploying two neurotechnology systems for different purposes (recording spinal responses to stimulation, or controlling an ‘experimental’ stimulation IC). We believe open access to xDev will lower the obstacles facing the development of future neurotechnology systems.

## Data Availability

The data that support the findings of this study are openly available at the following URL/DOI: https://github.com/neuromotion/xDev_manuscript.
